# Heterometallic cobalt(ii) calix[6 and 8]arenes: synthesis, structure and electrochemical activity†

**DOI:** 10.1039/d2ra01009g

**Published:** 2022-04-14

**Authors:** Anna Ignaszak, Nigel Patterson, Connor O'Brien, Allison True, Mark R. J. Elsegood, Timothy J. Prior, Carl Redshaw

**Affiliations:** Department of Chemistry, University of New Brunswick 30 Dineen Drive Fredericton NB E3B 5A3 Canada; Chemistry Department, Loughborough University Loughborough Leicestershire LE11 3TU UK; Department of Chemistry, University of Hull Cottingham Road Hull HU6 7RX UK c.redshaw@hull.ac.uk

## Abstract

Heterometallic cobalt *p-tert*-butylcalix[6 and 8]arenes have been generated from the *in situ* reaction of lithium reagents (*n*-BuLi or *t*-BuOLi) or NaH with the parent calix[*n*]arene and subsequent reaction with CoBr_2_. The reverse route, involving the addition of *in situ* generated Li[Co(O*t*-Bu)_3_] to *p-tert*-butylcalix[6 and 8]arene, has also been investigated. X-ray crystallography reveals the formation of complicated products incorporating differing numbers of cobalt and lithium or sodium centers, often with positional disorder, as well as, in some cases, the retention of halide. The electrochemical analysis revealed several oxidation events related to the subsequent oxidation of Co(ii) centers and the reduction of the metal cation at negative potentials. Moreover, the electrochemical activity of the phenol moieties of the parent calix[*n*]arenes resulted in dimerized products or quinone derivatives, leading to insoluble oligomeric products that deposit and passivate the electrode. Preliminary screening for electrochemical proton reduction revealed good activity for a number of these systems. Results suggest that [Co_6_Na(NCMe)_6_(μ-O)(*p-tert*-butylcalix[6]areneH)_2_Br]·7MeCN (6·7MeCN) is a promising molecular catalyst for electrochemical proton reduction, with a mass transport coefficient, catalytic charge transfer resistance and current magnitude at the catalytic turnover region that are comparable to those of the reference electrocatalyst (Co(ii)Cl_2_).

## Abstract 2

Reactions between *p-tert*-butylcalix[6 and 8]arenes and lithium or sodium reagents led to complex structures often with positional disorder. Such systems are capable of electrochemical proton reduction.

## Graphical abstract

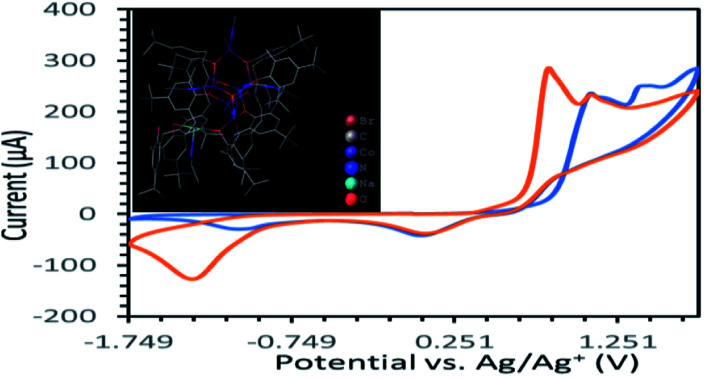

## Introduction

With a view to tackling future energy demands, there is currently immense interest in the design of catalysts capable of water splitting, thereby generating hydrogen. A number of metals have shown promise, but it is clear from the literature that the nature of the ligand set is equally important.^1–5^ Calix[*n*]arenes have demonstrated extensive metal coordination chemistry and their use in a variety of catalytic processes is well documented, although most of the reported work to-date utilizes *p-tert*-butylcalix[4]arene.^6–8^ We note however, that their use as ligands in water splitting systems is scant. Reports include Ti_4_ and Ti_6_ clusters based on thiacalix[4]arene scaffolds for the visible-light photocatalytic production of hydrogen, as well as polyoxotitanate clusters bearing calix[8]arene scaffolds which displayed remarkable H_2_ evolution ability.^9–11^ Noll and Würthner have also recently reported a dinuclear ruthenium complex bearing a functionalized calix[4]arene-based scaffold, which proved highly active in photocatalytic water oxidation.^12^

**Chart 1:**
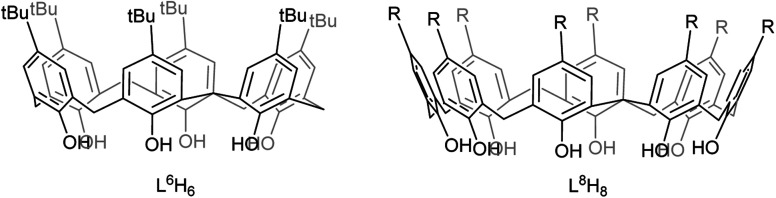
Calixarenes used in this study (R = H, *t*Bu).

**Chart 2:**
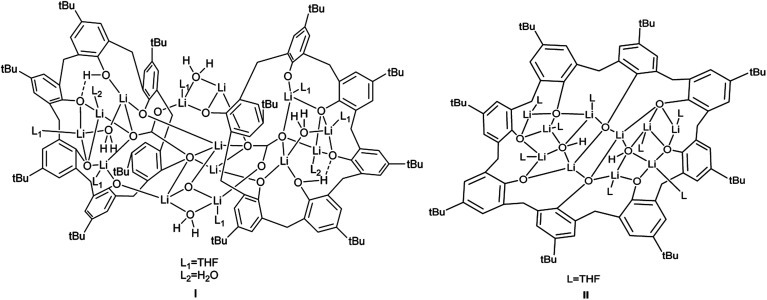
Multimetallic lithiated calix[*n*]arenes I and II formed on reaction with LiO*t*Bu (*n* = 6 (I), 8 (II)).

With this in mind, we have embarked upon a programme to investigate the electrochemical activity of metallocalix[*n*]arenes. Herein, our entry point into such chemistry is *via* the use of alkali metal calix[6 and 8]arenes, which can be used *in situ* and serve as a convenient point into mixed-metal calix[*n*]arene systems. The reverse strategy of generating a mixed-metal reagent and combining with the parent calix[*n*]arene can also provide an entry point into new metallocalix[*n*]arene chemistry.^13,14^ Our studies target new earth-abundant metallocalix[*n*]arenes, particularly those containing key metals that have already shown potential for water splitting in other systems.^1–5^ We find that it is possible to generate active cobalt/lithium-containing calix[6 and 8]arenes in one-pot *via* combinations of the parent calix[*n*]arene, cobalt(ii) bromide, and a lithium reagent, namely *n*-BuLi (*n*-butyllithium) or *t*-BuOLi (lithium *tert*-butoxide). We note that only a limited number of cobalt calix[6 and 8]arenes have been reported,^15,16^ whilst molecular cobalt species have shown promise as proton reduction catalysts.^17–20^ Moreover, we have recently reported the products I and II arising from the interaction of *p-tert*-butylcalix[6]areneH_6_ (L^6^H_6_) and *p-tert*-butylcalix[8]areneH_8_ (L^8^H_8_) with LiO*t*Bu, respectively, see Charts 1 and 2.^21^ Given the nature of the products formed herein, determination of the molecular structures by X-ray crystallography proved challenging and sometimes problematic. However, we note that structural characterization of metallocalix[*n*]arenes is considered to be towards the large end of ‘small molecule crystallography’, and the structures of such species are known the be inherently difficult to elucidate crystallographically with a high degree of precision.^22^ Nonetheless, this work highlights the electro-catalytic potential of cobalt/lithium species generated in the presence of large calix[*n*]arenes (*n* = 6, 8), and the molecular structure determinations highlight the complex nature of the heterometallic Li/Co systems employed herein.

## Experimental

### General experimental

All manipulations were performed under N_2_ using standard Schlenk techniques and dried, deoxygenated solvents. Acetonitrile was refluxed over calcium hydride. THF and diethylether were dried over sodium benzophenone. The ligands *p-tert*-butylcalix[6]areneH_6_ and *p-tert*-butylcalix[8]areneH_8_ were obtained from TCI UK and were dried *in vacuo* at 80 °C for 6 h prior to use. CoBr_2_ was purchased from Sigma Aldrich and was dried for 12 h at 100 °C *in vacuo* (affording the green form) prior to use. IR spectra (nujol mulls, KBr windows) were recorded on a Nicolet Avatar 360 FT IR spectrometer. Elemental analyses were performed by the elemental analysis service at the Department of Chemistry, the University of Hull. Mass spectra were recorded as LCMS, on a Bruker Daltonics HCT Ultra which uses Hystar 3.2. Data was processed in Data Analysis 4.2. For each sample, a 1 mg mL^−1^ stock solution was prepared, which was serially diluted to 10 μg mL^−1^ in dry acetonitrile. Example spectra are shown in Fig. S1 (of 6) and S2 (of 7) in the ESI.†

#### Preparation of {(*p-tert*-butylcalix[6]arene-Co-Li_4_(MeCN)_2_*t*-BuO)-CoBr_2_-(*p-tert*-butylcalix[6]arene-Co-Li_5_(MeCN)_5_THF)}·15MeCN (1·15MeCN)

Reagent ratio (L^6^H_6_ + 8LiO*t*Bu) + 2CoBr2.

A solution of lithium *tert*-butoxide (8.22 mL, 1.0 M in THF, 8.22 mmol) was added to *p-tert*-butylcalix[6]areneH_6_ (1.00 g, 1.03 mmol) in THF (30 mL) at ambient temperature. After stirring for 2 h, CoBr_2_ (0.45 g, 2.1 mmol) was added at −78 °C, and the system was stirred as it warmed up to ambient temperature. After stirring for 12 h, volatiles were removed *in vacuo*, and the residue was extracted, *via* filtration, into acetonitrile (30 mL). Prolonged standing (1 week) at 0 °C afforded light blue crystals of 1. Yield: 1.13 g, 65%. The sample was dried *in vacuo* for 12 h, which removed 20MeCN to afford {(*p-tert*-butylcalix[6]arene-Co-Li_4_(MeCN)*t*-BuO)-CoBr_2_-(*p-tert*-butylcalix[6]arene-Co-Li_5_(MeCN)THF)} (C_144_H_179_N_2_O_16_Li_9_Br_2_Co_3_) requires C 67.07, H 7.00, N 1.09%; found C 67.36, H 6.99, N 0.71%. IR (cm^–1^): 2299w, 2271w, 1602w, 1564w, 1364s, 1298s, 1261s, 1205s, 1170m, 1120m, 1101m, 1025m, 910m, 871m, 821s, 807s, 764m, 755m, 739m, 723m, 694w, 670w. M.S. (LCMS): 1451 (M–[6]H_6_–22MeCN–THF), 1358 (M–Co–5Li–[6]H_6_–22MeCN–THF), 1319 (M–Co–*t*BuO–[6]H_6_–22MeCN–THF).

#### Preparation of [Co_2_Li_8_Br_2_(OH)_2_(*p-tert*-butylcalix[8]arene)(NCMe)_4_(THF)_6_]·5MeCN·THF (2·5MeCN·THF)

Reactant ratio (L^8^H_8_ + 8LiO*t*Bu) + 2.2CoBr_2_.

As for 1, but using *p-tert-*butylcalix[8]areneH_8_ (1.00 g, 0.77 mmol) and LiO*t*-Bu (8.01 mL, 1.0 M solution in THF, 8.01 mmol) with CoBr_2_ (0.37 g, 1.7 mmol) affording 2 as blue prisms. Yield: 0.92 g, 47%. The sample was dried *in vacuo* for 2 h which removed 4MeCN and a THF to afford [Co_2_Li_8_Br_2_(OH)_2_(*p-tert*-butylcalix[8]arene)(NCMe)_4_(THF)_6_]·MeCN (C_122_H_169_N_5_O_16_Li_8_Br_2_Co_2_) requires C 63.85, H 7.42, N 3.05%; found C 63.80, H 7.22, N 2.43%. IR (cm^–1^): 3376bm, 3181w, 2298w, 2267w, 1745w, 1659m, 1606m, 1366s, 1295s, 1261s, 1203s, 1169s, 1152m, 1098s, 1046m, 966w, 906m, 875m, 819s, 803m, 760m, 738m, 722m, 664w, 637w, 553m, 533m, 476m, 439m. M.S. (LCMS): 1556 (M–7THF–9MeCN–Br–3Li), 1487 (M–7THF–9MeCN–Br–8Li–2OH).

#### Preparation of [*p-tert*-butylcalix[8]areneLi_3_Co_3_Br_2_(OH)_4_(Li(MeCN))_2_Li(MeCN)_3_Li(MeCN)(OH)(THF)]_2_[*p-tert*-butylcalix[8]areneLi_5_Co_3_(OH)_6_Br_2_Li_2_(MeCN)_4_(OH)_2_]·28MeCN (3·28MeCN)

Reactant ratio (L^8^H_8_ + 10.4LiO*t*Bu) + 3.1CoBr_2_.

As for 1, but using *p-tert-*butylcalix[8]areneH_8_ (1.00 g, 0.77 mmol) and LiO*t*-Bu (8.01 mL, 1.0 M solution in THF, 8.01 mmol) with CoBr_2_ (0.51 g, 2.3 mmol) affording blue prisms. Yield: 0.68 g, 43%. The sample was dried *in vacuo* for 12 h, which removed −20MeCN affording [*p-tert*-butylcalix[8]areneLi_3_Co_3_Br_2_(OH)_4_(Li(MeCN))_2_Li(MeCN)_3_Li(MeCN)(OH)(THF)]_2_[*p-tert*-butylcalix[8]areneLi_5_Co_3_(OH)_6_Br_2_Li_2_(MeCN)_4_(OH)_2_]·8MeCN (C_320_H_410_N_14_O_44_Li_21_Br_6_Co_9_) requires C 59.66, H 6.42, N 3.04%; found C 59.30, H 6.88, N 2.94%. IR (cm^–1^): 3549w, 2298w, 2270w, 1749w, 1660w, 1559w, 1482s, 1361s, 1313s, 1295s, 1261s, 1202s, 1169w, 1145w, 1098m, 1045s, 1025m, 963w, 930w, 906w, 880m, 874m, 820s, 809s, 760m, 739m, 723w, 710w, 673w, 664w, 618w, 553m, 534m, 519w, 471m, 440w. M.S. (LCMS): 1508 (M–2THF–42MeCN–4Br–21Li–18OH–8Co–2[8]), 1476 (M–2THF–42MeCN–5Br–19Li–16OH–8Co–2[8]).

#### Preparation of [*p-tert*-butylcalix[8]areneCo_3.2_Li_4.8_Br_2.73_(OH)_3.7_(MeCN)_1.6_]·9.5MeCN (4·9.5MeCN)

Reactant ratio (L^8^H_8_ + 8*n*-BuLi) + 4CoBr_2_.

To *p-tert*-butylcalix[8]areneH_8_ (1.00 g, 0.77 mmol) in diethylether (30 mL) at −78 °C was added *n*-BuLi (3.85 mL, 1.6 M in hexanes, 6.16 mmol) and the system was allowed to warm to ambient temperature and stirred for 12 h. The system was then cooled again to −78 °C and solid CoBr_2_ (0.67 g, 3.1 mmol) was added. After stirring for 12 h, volatiles were removed *in vacuo* and the residue was extracted, *via* filtration, into MeCN (30 mL). Prolonged standing (1 week) at 0 °C afforded 4 as blue needles. Yield: 1.08 g, 61%. The sample was dried *in vacuo* for 3 h, which removes 4.5MeCN to afford [*p-tert*-butylcalix[8]areneCo_3.2_Li_4.8_Br_2.7_(OH)_3.7_(MeCN)_1.6_]·5MeCN (C_111.2_H_139.3_N_11.1_O_11.67_Li_4.8_Co_3.2_Br_2.7_) requires C 58.96, H 6.14, N 4.19%; found C 58.03, H 6.73, N 3.59%. IR (cm^–1^): 3422bm, 2299w, 2270w, 1614w, 1479s, 1414m, 1391m, 1361s, 1295s, 1261s, 1204s, 1145m, 1097s, 1019s, 967w, 907w, 875m, 820m, 802s, 762w, 739w, 722w, 709w, 665w. M.S. (LCMS): 1363 (M–11.1MeCN–2.73Br–2.8Li–3.7OH–2.2Co).

#### Preparation of [Co_2_Li_6_Br_2_(OH)_2_(*p-tert*-butylcalix[8]arene)(NCMe)_4_]·16.5MeCN (5·16.5MeCN)

Reactant ratio (CoBr_2_ + 3LiO*t*Bu) + ⅓L^8^H_8_.

To CoBr_2_ (0.68 g, 3.1 mmol) in diethylether (40 mL) at −78 °C was added LiO*t*-Bu (9.33 mL, 1.0 M in THF, 9.33 mmol) and the system was allowed to warm to ambient temperature and stirred for 12 h. The system was then cooled again to −78 °C and *p-tert*-butylcalix[8]areneH_8_ (1.34 g, 1.03 mmol) in THF (40 mL) was added. Following stirring for 12 h at ambient temperature, the volatiles were removed *in vacuo* and the residue was extracted, *via* filtration, into MeCN (30 mL). Standing for 2–3 days at ambient temperature afforded 5 as a blue/green crystalline solid. Yield: 1.08 g, 42%. The sample was dried *in vacuo* for 12 h, which removed 17.5MeCN to afford [Co_2_Li_6_Br_2_(OH)_2_(*p-tert*-butylcalix[8]arene)(NCMe)_3_] (C_96_H_115_Br_2_O_10_Co_2_Li_6_N_3_) requires 65.28, H 6.56, N 2.34%; found C 65.96, H 6.66, N 2.66%. IR (cm^–1^): 3398 bs, 2374w, 2297w, 2269w, 2253w, 1636m, 1361s, 1311m, 1294s, 1263m, 1205s, 1155w, 1120w, 1098w, 1050w, 1023m, 972w, 910m, 881w, 869w, 820m, 807w, 800w, 767w, 755w, 721w, 704w, 689w, 665w, 639w, 615w, 545m, 531, 505w, 488w, 470w, 451w. M.S. (LCMS): 1407 (M–20.5MeCN–2Br–OH–Co), 1390 (M–20.5MeCN–2Br–2OH–Co), 1376 (M–20.5MeCN–2Br–2OH–Co–Li).

#### Preparation of [Co_6_Na(NCMe)_6_(μ-O)(*p-tert*-butylcalix[6]areneH)_2_Br]·7MeCN (6·7MeCN)

Reactant ratio (L^6^H_6_ + 6NaH) + 3CoBr_2_.

To *p-tert*-butylcalix[6]areneH_6_ (1.00 g, 1.03 mmol) in THF (30 mL) was added NaH (0.15 g, 6.2 mmol) and the system was refluxed for 12 h. The system was then cooled to −78 °C and CoBr_2_ (0.68 g, 3.1 mmol) was added, and the system was slowly allowed to warm to room temperature and stirred for 12 h. After which, volatiles were removed *in vacuo*, and the residue was extracted, *via* filtration, into MeCN (30 mL). Small light blue prisms of 6·7MeCN formed on standing (1–2 days) at room temperature (15 °C). Yield: 1.07 g, 70%. The sample was dried *in vacuo* for 12 h, which removed −2MeCN affording [Co_6_Na(NCMe)_6_(μ-O)(*p-tert*-butylcalix[6]areneH)_2_Br]·5MeCN (C_154_H_191_BrO_13_Co_6_NaN_11_) requires C 64.65, H 6.73, N 4.90%; found C 65.62, H 6.97, N 5.19%. IR (cm^–1^): 2312m, 2284m, 1607w, 1365s, 1294m, 1260s, 1201s, 1094m, 1024s, 944w, 910w, 872w, 801s, 723m, 684w, 522w, 486w, 430w. M.S. (LCMS): 1046 (M–13MeCN–Br–Na–5Co–[6]H_2_), 1030 (M–13MeCN–Br–Na–5Co–O–[6]H_2_).

#### Preparation of [Co_4_Na(NCMe)_6_(μ-O)(*p-tert*-butylcalix[8]arene)(*p-tert*-butylcalix[8]areneH_5_)Br]·13MeCN (7·13MeCN)

Reactant ratio (L^8^H_8_ + 8NaH) + 4CoBr_2_.

As for 6, but using *p-tert*-butylcalix[8]areneH_8_ (1.00 g, 0.77 mmol), NaH (0.15 g, 6.3 mmol) and CoBr_2_ (0.68 g, 3.1 mmol). Yield: 0.83 g, 65%. A freshly prepared batch of crystals was found to contain 20 solvent molecules of crystallization: [Co_4_Na(NCMe)_6_(μ-O)(*p-tert*-butylcalix[8]arene)(*p-tert*-butylcalix[8]areneH_5_)Br]·20MeCN (C_228_H_291_BrO_16_Co_4_NaN_26_) requires C 68.62, H 7.35, N 9.13%; found C 68.20, H 7.67, N 9.11%. IR (cm^–1^): 3539w, 3483w, 2309w, 2278w, 2250w, 1610w, 1566w, 1364s, 1298s, 1207s, 1154w, 1120m, 1022s, 935m, 906m, 875m, 818s, 802s, 748m, 723m, 637w, 548w, 528w. M.S. (LCMS): 2315 (M–*p-tert*-butylcalix[8]arene–Na–O–Co).

### Electrochemical methods

Dry acetonitrile was obtained from the solvent purification system (UNB, Fredericton, Canada). Silver nitrate (ACS reagent, ≥ 99.0%) and tetraethylammonium tetrafluoroborate, TEABF_4_ (99%) and glacial acetic acid (ReagentPlus®, ≥99%) were obtained from Sigma Aldrich, Canada. All electrochemical experiments were carried out on a PalmSense electrochemical workstation, using an electrochemical cell equipped with a platinum wire counter electrode, a non-aqueous Ag/Ag^+^ reference electrode (0.681 V *vs.* SHE (standard hydrogen electrode), prepared by filling the electrode with a solution of 0.01 M AgNO_3_/0.1 M TEABF_4_ in dry CH_3_CN), and the glassy carbon disk electrode with 0.1963 cm^2^ area used as a working electrode. The concentration of cobalt calix[6 and 8]arenes dissolved in the electrolyte was 0.25–1 mM. The electrochemical activity for the hydrogen evolution reaction (HER) was carried out for 0–2 mM of glacial acetic acid, added to the electrochemical cell in 0.2 mM aliquots. The CV scans were acquired over the potential range from −1.75 to +1.75 V (*vs.* Ag/Ag^+^) with a scan rate of 0.05–20.0 V s^−1^. The electrochemical stability of the catalysts was tested in the absence and presence of protons (at 2 mM of glacial acetic acid) by scanning the potential from −1.75 to +1.75 V (*vs.* Ag/Ag^+^) at 0.15 V s^−1^ (typically 20 CV scans). AC electrochemical impedance spectroscopy was used for the HER kinetic studies within frequency range 0.1–10^5^ Hz at a potential amplitude of 10 mV at the following constant polarization: open circuit potential (OCP), at *E*_onset_ and *E*_peak_ for the HER reaction in the absence and presence of 2 mM acetic acid. Differential pulse voltammetry (DPV) was applied in order to improve the peak separation in the potential range adjusted to particular type of calixarenes. A potential step of 0.004 V, a pulse width of 0.06 s, a pulse period of 0.5 s, and a pulse amplitude of 0.05 V were used in all DPV experiments. The electrode surface was cleaned by polishing using an alumina slurry (Al_2_O_3_ particle size of 0.05 μm; supplied by Pine Research Instrumentation).

### Crystal structure determinations

Diffraction data were collected on a variety of modern diffractometers equipped with CCD, hybrid pixel array, or image plate detectors. X-ray sources were either conventional or micro-focus sealed tubes or rotating anodes generating either Mo-Kα or Cu-Kα X-radiation. Full details are presented in Table 2 and in the deposited cif files. Structures were solved and refined routinely^23^ except as shown in ref. 22. Regions of disordered solvent were modelled using the SQUEEZE routine.^24^

**Table 1:** Diffusion coefficient (*D*, cm s^−1^); *E*_onset_ and *E*_peak_ for electrochemical proton reduction; overpotential of proton reduction (*η*, V); and charge transfer resistance recorded at the constant potential in the catalytic turnover region (*R*_3_, ohms)

Catalyst	*D* a (cm s^−1^)	*E*_onset_ b (V)	*E*_peak_ (V)	*η*_HER_ c (mV)	*R*_3_ (ohms) at *E*_peak_
Co(ii)Cl_2_	1.13 × 10^−9^	−0.695	−1.279	14	1234
6^d^	3.33 × 10^−10^	−0.711	−1.333	30	6980
7^e^	1.01 × 10^−10^	−0.822	−1.533	141	28 690

a (*D* estimated for 1 mM solutions in electrolyte).

b *E vs.* non-aqueous reference electrode Ag/Ag^+^
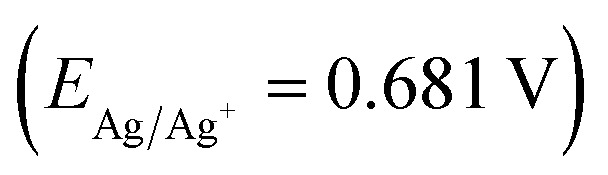
 for 1 mM catalyst in electrolyte (CV demonstrated in Fig. 10).

c *η*_HER_ = *E*^0^_HER_ − *E*; where *n* is overpotential defined as a difference between the potential at which electrode is operated and the standard potential of the formation of H_2_ from H^+^ at the Pt electrode in aqueous electrolyte (H^+^ + e^−^ = 1/2H_2_ at 0.0 V *vs.* standard hydrogen electrode or −0.681 V *vs.* Ag/Ag^+^ used in this work).

d [Co_6_Na(NCMe)_6_(μ-O)(*p-tert*-butylcalix[6]areneH)_2_Br]·7MeCN (6·7MeCN).

e [Co_4_Na(NCMe)_6_(μ-O)(*p-tert*-butylcalix[8]arene)(*p-tert*-butylcalix[8]areneH_5_)Br]·13MeCN (7·13MeCN).

X-ray crystallographic data

**Table:** 

Compound	1·15MeCN	2·5MeCN·THF	3·28MeCN
Formula	C_184_H_239_Br_2_O_16_Co_3_Li_9_N_22_	C_134_H_189_Br_2_O_17_Co_2_Li_8_N_9_	C_360_H_460_Br_6_O_44_Co_9_Li_21_N_44_
Formula weight	3399.72	2531.13	7263.26
Crystal system	Monoclinic	Triclinic	Monoclinic
Space group	*P*2_1_/*c*	*P*1̄	*P*2_1_/*n*

**Unit cell dimensions**			
*a* (Å)	16.6753(4)	10.6094(7)	10.4262(2)
*b* (Å)	61.6021(17)	17.1327(12)	57.4173(10)
*c* (Å)	18.8069(7)	20.9110(15)	35.8800(8)
*α* (°)	90	107.144(4)	90
*β* (°)	91.986(2)	93.425(3)	96.577(2)
*γ* (°)	90	104.202(4)	90
*V* (Å^3^)	19 307.5(10)	3485.1(4)	21 338.0(7)
*Z*	4	1	2
Temperature (K)	100(2)	100(2)	100(2)
Wavelength (Å)	1.54178	0.71073	0.71075
Calculated density (g cm^−3^)	1.102	1.206	0.958
Absorption coefficient (mm^−1^)	0.726	0.87	0.953
Transmission factors (min./max.)	0.712, 1.000	0.644, 1.000	0.7653, 1.000
Crystal size (mm^3^)	0.220 × 0.120 × 0.015	0.160 × 0.120 × 0.070	0.150 × 0.100 × 0.090
*θ*(max) (°)	25.0	27.5	25.027
Reflections measured	79 547	56 328	158 630
Unique reflections	79 547	15 899	37 692
*R*_int_	0.30	0.063	0.0539
Reflections with *F*^2^ > 2*σ*(*F*^2^)	79 547	10 729	24 480
Number of parameters	2006	917	1787
*R*_1_ [*F*^2^ > 2*σ*(*F*^2^)]	0.1766	0.114	0.1150
*wR*_2_ (all data)	0.4369	0.382	0.3406
GOOF, *S*	1.065	1.04	1.468
Largest difference peak and hole (e Å^−3^)	1.723 and −1.053	1.91 and −0.88	1.735 and −1.497

**Table:** 

Compound	4·9.5MeCN	5·16.5MeCN
Formula	C_110.2_H_139.3_Br_2.73_O_11.67_Co_3.2_Li_4.8_N_11.1_	C_129_H_167.5_Br_2_O_10_Co_2_Li_6_N_20.5_
Formula weight	2246.18	2484.65
Crystal system	Tetragonal	Tetragonal
Space group	*I*4_1_/*a*	*I*4_1_/*a*

**Unit cell dimensions**		
*a* (Å)	41.6832(5)	41.7784(4)
*b* (Å)	41.6832(5)	41.7784(4)
*c* (Å)	17.4818(3)	17.5681(3)
*α* (°)	90	90
*β* (°)	90	90
*γ* (°)	90	90
*V* (Å^3^)	30 374.4(9)	30 664.0(8)
*Z*	8	8
Temperature (K)	100(2)	100(2)
Wavelength (Å)	0.71075	0.71073
Calculated density (g cm^−3^)	0.866	1.076
Absorption coefficient (mm^−1^)	0.805	0.79
Transmission factors (min./max.)	0.9877, 1.011	0.391, 1.000
Crystal size (mm^3^)	0.150 × 0.050 × 0.050	0.190 × 0.160 × 0.120
*θ*(max) (°)	25.028	25.000
Reflections measured	170 311	84 206
Unique reflections	13 402	13 484
*R*_int_	0.1200	0.061
Reflections with *F*^2^ > 2*σ*(*F*^2^)	6984	10 320
Number of parameters	551	674
*R*_1_ [*F*^2^ > 2*σ*(*F*^2^)]	0.0968	0.086
*wR*_2_ (all data)	0.3319	0.291
GOOF, *S*	1.046	1.04
Largest difference peak and hole (e Å^−3^)	0.720 and −0.2452	0.64 and −0.44

**Table:** 

Compound	6·7MeCN	7·13MeCN
Formula	C_158_H_197_BrO_13_Co_6_NaN_13_	C_214_H_270_BrO_16_Co_4_NaN_19_
Formula weight	2942.75	3703.09
Crystal system	Triclinic	Monoclinic
Space group	*P*1̄	*P*2_1_/*n*

**Unit cell dimensions**		
*a* (Å)	16.9279(2)	21.7315(2)
*b* (Å)	21.3316(3)	31.5316(5)
*c* (Å)	24.0984(4)	29.9771(5)
*α* (°)	88.4380(10)	90
*β* (°)	85.3530(10)	94.9100(10)
*γ* (°)	68.1970(10)	90
*V* (Å^3^)	8052.8(2)	20 465.8(6)
*Z*	2	4
Temperature (K)	100(2)	100(2)
Wavelength (Å)	0.71075	1.54184
Calculated density (g cm^−3^)	1.213	1.082
Absorption coefficient (mm^−1^)	5.510	3.200
Transmission factors (min./max.)	0.56663, 1.0000	0.617, 1.000
Crystal size (mm^3^)	0.200 × 0.070 × 0.015	0.310 × 0.080 × 0.050
*θ*(max) (°)	68.245	55.991
Reflections measured	173 441	126 916
Unique reflections	29 268	26 621
*R*_int_	0.0878	0.0874
Reflections with *F*^2^ > 2*σ*(*F*^2^)	22 098	21 440
Number of parameters	1512	2013
*R*_1_ [*F*^2^ > 2*σ*(*F*^2^)]	0.0702	0.0940
*wR*_2_ (all data)	0.2115	0.2669
GOOF, *S*	1.061	1.037
Largest difference peak and hole (e Å^−3^)	1.330 and −1.630	1.63 and −0.52

The crystallographic determination of the structures presented here is difficult for a number of reasons. The first problem is the presence of large unit cells with significant regions occupied by disordered solvent. This leads to inherently weak scattering of X-rays. To overcome some of the difficulties of the weak scattering we have employed the brightest laboratory sources with modern detectors at the UK National Crystallography Service. Frequently, small-scale disorder is also a problem and this has been treated conservatively using standard methods. For the majority of structures it has not been possible to locate hydrogen atoms and these are placed at calculated positions. The challenge in locating hydrogen atoms means the assignment of hydroxide or water is very difficult.

The determination of the metals present is also difficult. In some cases (*e.g.* compound 1) the metals are in an ordered arrangement, but in many of the refinements it emerged that a significantly better fit to the observed data was obtained if the lithium and cobalt were allowed to share crystallographic sites. Where this is the case, the constitution of a particular site has been determined by allowing the amount of Li and Co to refine freely subject to the condition that the total occupancy is unity, and that the Li and Co have the same displacement parameter. This leads to average values for the occupancy of the sites within the whole crystal.

## Results and discussion

### Synthesis and molecular structures

#### Use of lithiated reagents

##### *p-tert*-Butylcalix[6]arene (L^6^H_6_) complex

**Fig. 1:**
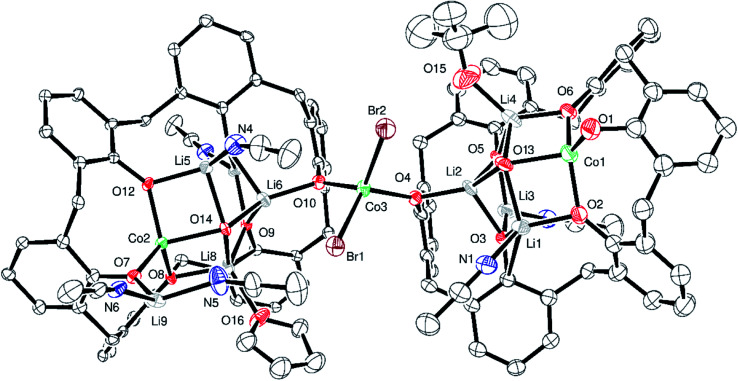
Asymmetric unit of 1 with atoms drawn as 30% probability ellipsoids. For clarity, unbound solvent, *t*-butyl groups of the calixarene, and hydrogen atoms are not shown. Selected bond lengths (Å) and angles (°): Co1–O1 1.916(14), Co1–O2 1.906(12), Co1–O6 1.910(12), Co1–O13 2.043(12), Co2–O7 1.935(8), Co2–O8 1.953(9), Co2–O12 1.917(9), Co2–O14 2.042(9), Co3–O4 1.907(9), Co3–O10 1.891(10), Co3–Br1 2.437(3), Co3–Br2 2.416(3); O1–Co1–O13 152.5(6), O2–Co1–O6 130.3(5), O7–Co1–O14 121.1(4), O8–Co2–O12 128.6(4), O4–Co3–O10 118.1(3), Br1–Co3–Br2 107.65(10).

The interaction of L^6^H_6_ with excess LiO*t*Bu has been shown to afford the complex [Li_14_(L^6^H)_2_(CO_3_)_2_(THF)_6_(OH_2_)_6_].^25^ We were interested if such a species could be employed as an entry point into heterometallic calixarene systems. With this in mind, L^6^H_6_ in THF was treated with excess LiO*t-*Bu (8 equiv.) at ambient temperature, and after 2 h, the system was cooled to −78 °C and CoBr_2_ (2 equiv.) was added. Work-up (extraction into MeCN) afforded the complex {(*p-tert*-butylcalix[6]areneCoLi_4_(MeCN)_2_*t-*BuO)CoBr_2_(*p-tert*-butylcalix[6]areneCoLi_5_(MeCN)_4_THF)}·15MeCN (1·15MeCN). There are 439 atoms in the asymmetric unit which consists of two metallated calix[6]arenes linked by a cobalt ion (bridging CoBr_2_ unit), see Fig. 1 (an alternative view is given in Fig. S3, ESI†). The structure is a twin and was refined using the HKLF5 formalism. It contains large solvent accessible voids and the data were treated using SQUEEZE (420 electrons recovered due to disordered solvent in the unit cell, included in formula above).^24^ There is a near square arrangement of metals and the metal ions are ordered (3 × Li, 1 × Co). In each case, over one end, there is an extra Li ion. The squares reside within the *p-tert*-butylcalix[6]arene. Solvent molecules complete the coordination of the metal ions. On one *p-tert*-butylcalix[6]arene, there is a further Li ion. Notably, the two calix[6]arenes are bridged by C–O–CoBr_2_–O–C units. There is 11% solvent-accessible volume centred on two pockets within the unit cell (at ½, 0, ½ and ½, ½, 1). In terms of charge, the 15− available from the two fully deprotonated calix[6]arene ligands, the 2 bromides and the *tert*-butoxide ligand, is balanced by the 3 cobalt(ii) centres and 9 lithium centres.

##### *p-tert*-Butylcalix[8]arene complexes

**Fig. 2:**
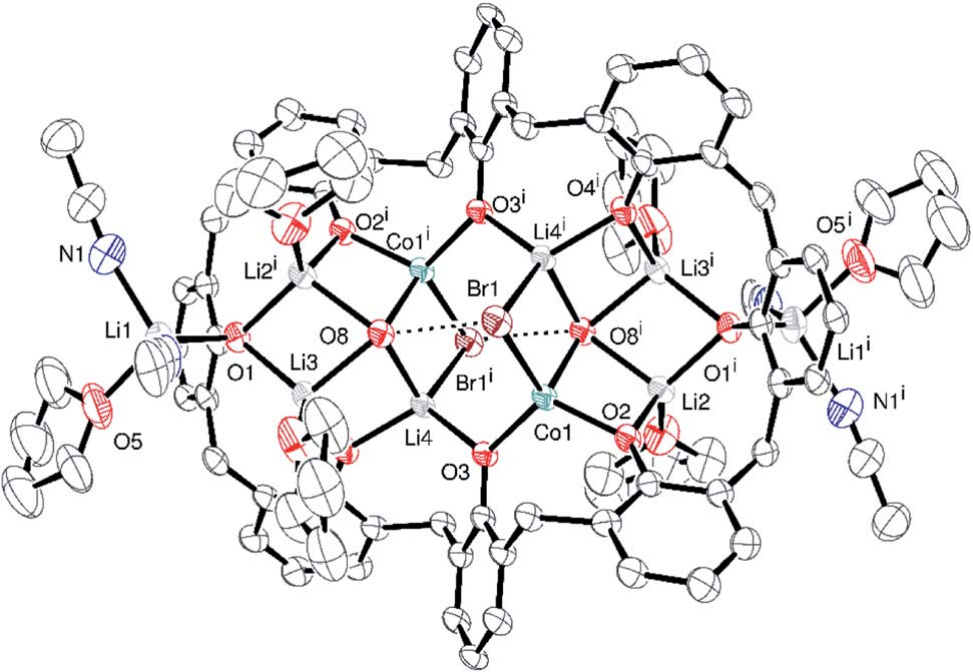
Molecular structure of [Co_2_Li_8_Br_2_(OH)_2_(*p-tert*-butylcalix[8]arene)(NCMe)_4_(THF)_6_]·5MeCN·THF (2·5MeCN·THF) with atoms as 30% probability ellipsoids. For clarity, *t*-butyl groups, the hydrogen atoms, and disorder are not shown. Dashed lines show intramolecular hydrogen bonds. Selected bond lengths (Å) and angles (°): Co1–O2 1.906(4), Co1–O3 1.937(3), Co1–O8A 2.044(4), Co1–Br1 2.4755(13); Li2–O1A 1.936(10), Li2–O2 1.976(10), Li2–O8A 2.048(10), Li2–O6 1.062(10) Br1⋯O8 3.456(3); Co1–Br1 Li4A 70.43(5), Co1–O2–Li2 92.2(3), Co1–O3–Li4 109.44(18), Co1–O8A–Li4A 89.64(16).

Reaction of L^8^H_8_ with excess LiO*t*Bu has been shown to afford [Li_10_(L^8^)(OH)_2_(THF)_8_].^25^ Herein, *in situ* reaction of the mixture generated from L^8^H_8_ with excess LiO*t-*Bu (10.4 equiv.) at ambient temperature, followed by the addition of CoBr_2_ (2.2 equiv.) led to the isolation of the complex [Co_2_Li_8_Br_2_(OH)_2_(*p-tert*-butylcalix[8]arene)(NCMe)_4_(THF)_6_]·5MeCN·THF (2·5MeCN·THF). Although the diffraction data was poor, with clear signs of twinning, the connectivity has been clearly established (Fig. 2; for alternative views, see Fig. S4, ESI†). The molecule lies on a centre of symmetry and half of the formula is in the asymmetric unit. One MeCN and the THF of crystallisation were modelled as a region of diffuse electron density *via* the PLATON Squeeze procedure.^24^ The Co(1) and Li(4) positions have positional disorder with part Co part Li at each site. At Co(1) its 62.8(3)% Co, and at Li(4) its 62.8(3)% Li and *vice versa*. Thus, one Co and one Li in total over these two sites. The Co is in the +2 oxidation state, assuming O(8) is a hydroxide; while an H atom could not be located on O(8), this O does make a plausible H-bond to Br(1A). In terms of charge, the 12− available from the fully deprotonated calix[8]arene ligand, the 2 bromides and 2 hydroxides, is balanced by 2 cobalt(ii) centres and 8 lithium centres.

**Fig. 3:**
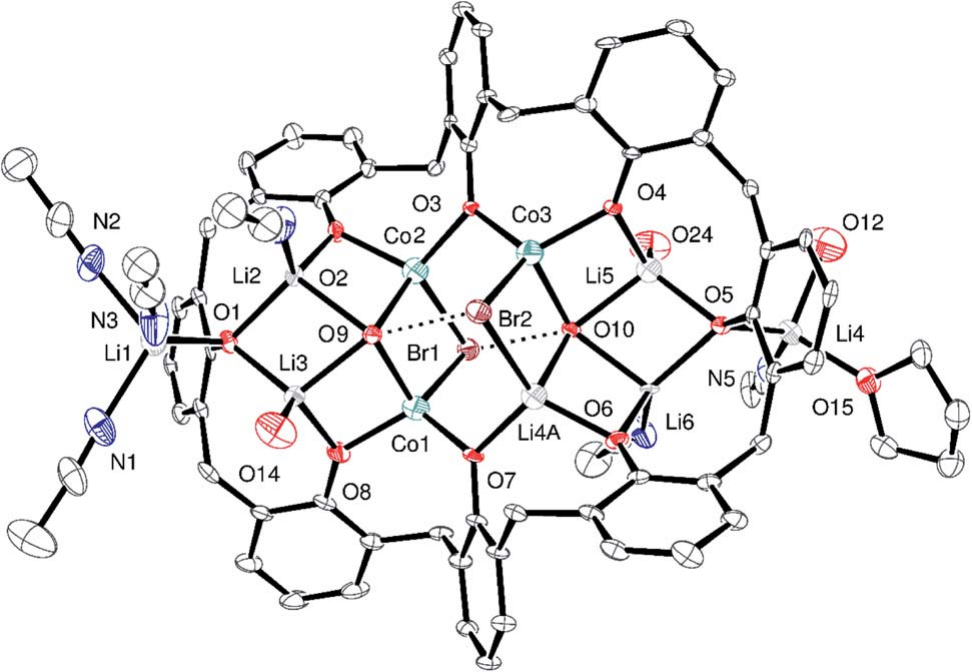
Major part of the asymmetric unit of 3·28MeCN with atoms shown as 50% probability ellipsoids. For clarity, *t*-butyl groups, non-coordinated solvent and hydrogen atoms are not shown. Dashed lines show hydrogen bonds. Selected bond lengths (Å): Li6–O6 1.836(10), Li6–O5 1.957(10), Li6–O10 2.017(11), Li6–N6 2.042(11), Li7–O17 1.844(12), Li7–O16 1.941(12), Li7–O21 2.018(13), Li7–O20 2.025(12), Li7–Co6 2.686(12), Li9–O16 1.905(15), Li9–N7 1.99(2), Li9–N8 2.018(18), Li9–O22 2.30(2), Li9–C112 2.662(15), Co1–O8 1.877(5), Co1–O7 1.925(5), Co1–O9 2.021(4), Co1–Br1 2.4714(14), Co2–O2 1.920(5), Co2–O3 1.925(5), Co2–O9 2.014(5), Co2–Br1 2.562(2), Co3–O3 1.905(4), Co3–O4 1.905(4), Co3–O10 2.039(4), Co3–Br2 2.4695(14), Li4A–O6 1.887(5), Li4A–O7 1.934(5), Li4A–O10 2.026(5), Li4A–Br2 2.568(2), Li5–O4 1.913(14), Li5–O5 1.932(15), Li5–O24 1.973(15), Li5–O10 2.022(14); O9⋯Br2 and O10⋯Br1 are 3.448(4) and 3.397(4) Å respectively; Co1–O9–Co2 90.70(19), Co1–Br1–Co2 69.53(6), Co2–O3–Co3 108.2(2).

Similarly, *in situ* reaction of the mixture generated from *p-tert-*butylcalix[8]areneH_8_ and LiO*t*-Bu (10.4 equiv.) with CoBr_2_ (3.1 equiv.) led to the isolation of crystals of composition [*p-tert*-butylcalix[8]areneLi_3_Co_3_Br_2_(OH)_4_(Li(MeCN))_2_Li(MeCN)_3_Li(MeCN)(OH)(THF)]_2_[*p-tert*-butylcalix[8]areneLi_5_Co_3_(OH)_6_Br_2_Li_2_(MeCN)_4_(OH)_2_]·28MeCN (3·28MeCN). The crystal structure is complicated because of the size and complexity of the asymmetric unit (Fig. 3; for an alternative view, see Fig. S5, ESI†), the presence of disorder, and unresolved solvent. The asymmetric unit contains one and a half calix[8]arene molecules and associated metals; there are 394 atoms in the asymmetric unit. In this structure, there is limited disorder on the metal sites, and there are two different types of *p-tert*-butylcalix[8]arene present in a 2 : 1 ratio. Each *p-tert*-butylcalix[8]arene binds two sets of four metal ions, with the metals forming a square capped by hydroxide; note O12 is also OH^−^. There is additionally a lithium cation attached to each cluster together with THF/MeCN. In terms of charge in 3·28MeCN, if Co(iii) have +48 from 9 Co plus 21 Li, which is balanced by 6 Br, 18 OH and 3 L^8^.

There is a single, huge pocket of 34% of the volume of the unit cell centred on 0.245, 0.252, 0.245. Adjacent pockets are interconnected to give solvent accessible channels that extend in the *yz* plane (Fig. S6, ESI†). The solvent was modelled using SQUEEZE;^24^ the unresolved electron density corresponds to 10 extra solvent (MeCN) molecules in the unit cell.

**Fig. 4:**
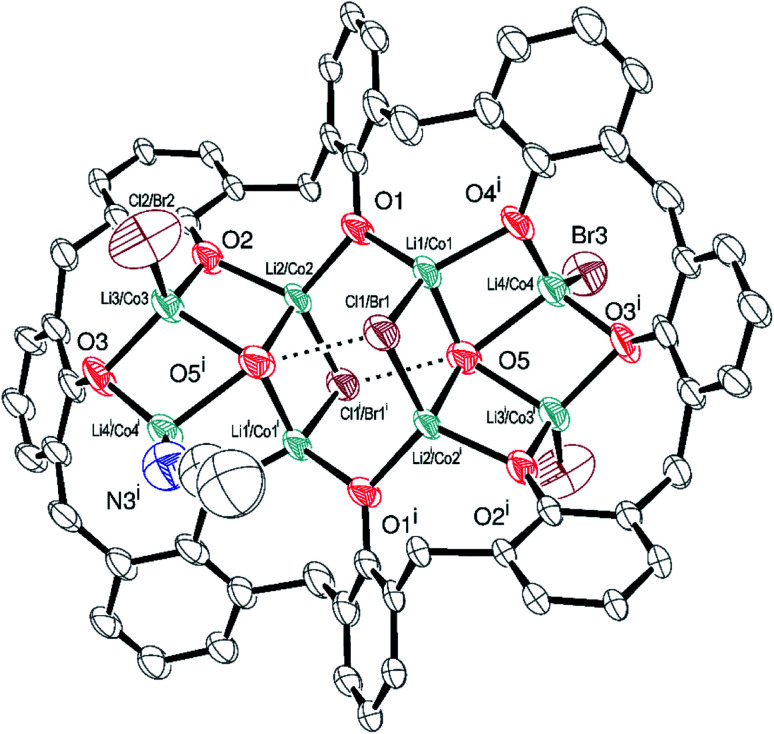
ORTEP representation of [*p-tert*-butylcalix[8]areneCo_3.2_Li_4.8_Br_2.73_(OH)_3.7_(MeCN)_1.6_]·9.5MeCN (4·9.5MeCN) with atoms drawn as 30% probability ellipsoids. The full calixarene shown above is generated by the symmetry operator *i* = 1 − *x*, 1 − *y*, 1 − *z*. For clarity, *t*-butyl groups, hydrogen atoms, disorder, and a single (ordered) acetonitrile molecule are not shown. Dashed lined show hydrogen bonds. Selected bond lengths (Å) and angles (°): selected bond lengths (Å): Br3–Co4 2.442(13), O1–Co2 1.922(4), O1–Co1 1.928(4), O2–Co3 1.851(5), O2–Co2 1.916(4), O3–Co4^i^ 1.797(7), O3–Co3 1.908(4), O4–Co4^i^ 1.877(8), O4–Co1^i^ 1.921(4), O5–Co3^i^ 2.013(5), O5–Co2^i^ 2.053(5), O5–Co1 2.066(5), O5–Co4 2.156(6), Co1–Br1 2.4907(18), Co1–Co4 2.769(6), Co1–Co2^i^ 2.829(2), Co2–Br1^i^ 2.511(3), Co2–Co3 2.715(3), Co3–Br2 2.339(4), Co3–Co4^i^ 2.414(5); O5^i^⋯Br1 = 3.454 Å; Co1–O1–Co2 110.2(2), O3–Co3–O5^i^ 102.6(2).

On generating a lithiated *p-tert*-butylcalix[8]arene *via* the use of *n*-BuLi and subsequent addition of CoBr_2_, the product isolated upon work-up was found to be the complex [*p-tert*-butylcalix[8]areneCo_3.2_Li_4.8_Br_2.73_(OH)_3.7_(MeCN)_1.6_]·9.5MeCN (4·9.5MeCN). As in the previous complex, the calix[8]arene binds two sets of four metal ions (Fig. 4; for an alternative view, see Fig. S7, ESI†), with each square capped by a hydroxide. The situation is complicated by mixed Co/Li at the metal sites. There is some disorder too as bromide/acetonitrile share one coordination site. The calix[8]arenes are packed to generate huge channels in the structure running parallel to the crystallographic *c*-axis (Fig. S8, ESI†). These occupy nearly 40% of the crystal structure and are filled by disordered solvent. Scattering from this disordered solvent was modelled using SQUEEZE.^24^ A total of 12 MeCN per formula unit were identified by this method and are included in the formula above.

In terms of charge, there is 14.2− available from the fully deprotonated calix[8]arene ligand, the 2.62 chlorides, 1.58 bromides and 2 hydroxides. The geometric parameters don't allow us to distinguish between Co(ii) and Co(iii) for this system. The presence of 2 cobalt(ii) centres and 5.19 lithium centres here would afford +10.81, whilst 2 Co(iii) centres would lead to +13.62. In either case, partial protonation of either L^8^ or OH is required to off-set the charge; there is no firm evidence for protonation.

**Fig. 5:**
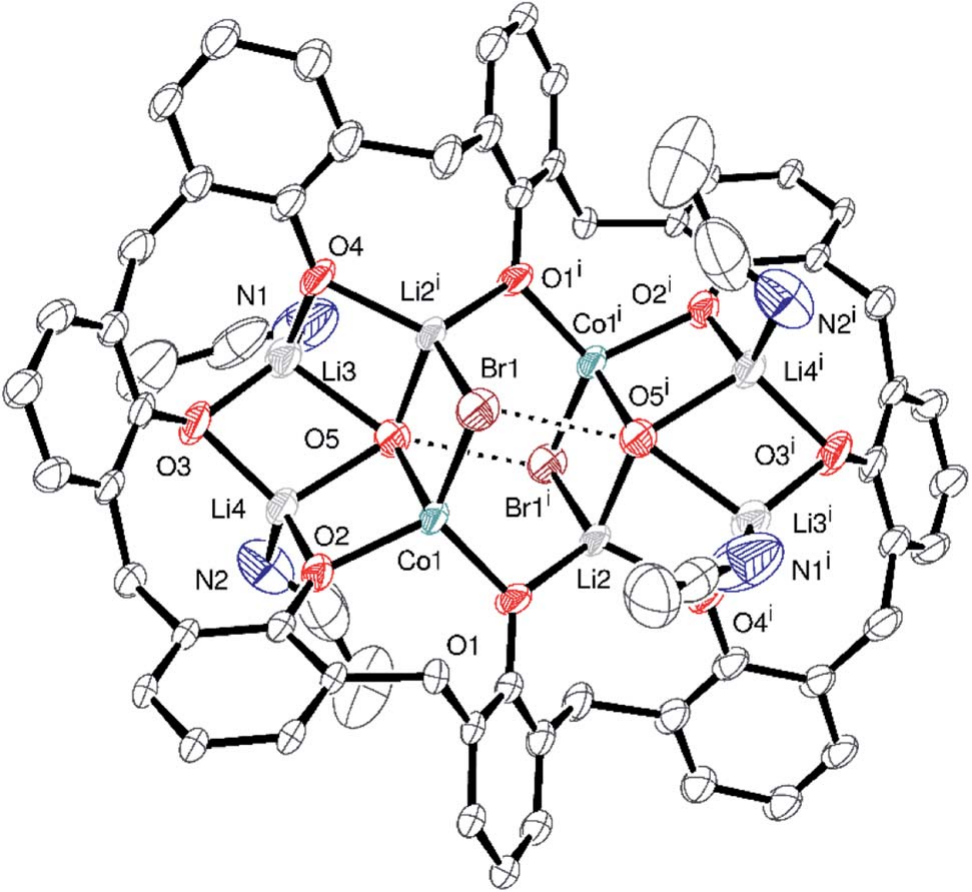
Molecular structure of [Co_2_Li_6_Br_2_(OH)_2_(*p-tert*-butylcalix[8]arene)(NCMe)_4_]·16.5MeCN (5·16.5MeCN) with atoms as 30% probability ellipsoids. For clarity, *t*-butyl groups, hydrogen atoms, disorder and acetonitrile molecules are not shown. Dashed lines show the positions of hydrogen bonds. Selected bond lengths (Å) and angles (°): Co1–O1 1.916(1), Co1–O2 1.959(3), Co1–O5 2.046(4), Co1–Br1 2.4592(17), Li2–O4^i^ 1.962(3), Li2–O5^i^ 2.084(4), Li3–O3 1.790(6), Li3–O5 2.152(6); O1–Co1–O2 107.97(16), Co1–Br1–Li4A 69.56(5). Symmetry operator used to generate equivalent atoms: *i* = (1 − *x*, 1 − *y*, 1 − *z*).

The reverse addition, in which the salt Li[Co(O*t*-Bu)]_3_, generated *in situ via* an adaptation of the Wilkinson method,^25^ was added to *p-tert*-butylcalix[8]arene, led to the isolation of the complex [Co_2_Li_6_Br_2_(OH)_2_(*p-tert*-butylcalix[8]arene)(NCMe)_4_]·16.5MeCN (5·16.5MeCN), see Fig. 5. Half of this is the asymmetric unit, whilst each of the four unique metal sites is part Li and part Co; *i.e*. there is site disorder. The formula stated is based on the most likely composition given that Li is 1+ and Co is 3+, plus the evidence from free refinement of the occupancies of both metals at each site (Table S2, ESI†). The structure was refined with occupancies at each site allowed to vary freely. The total positive charge based on the refined occupancies is Co: 7.118 + Li: 5.604 = 12.722. In order to make sense chemically, the formula was assumed to be Co_2_Li_6_ giving (2 × 3+) + (6 × 1+) = 12+ in total. The calixarene is fully deprotonated, O(5) is OH^−^, but the H atom could not be located. This would make a good H-bond to the Br(1), as seen previously, with an O⋯Br distance of 3.456(3) Å. To counter balance the metals present, the negative charges are 8− for the calixarene, 2 × 1− for the OHs, and 2 × 1− for the bromides, *i.e.* 12−. Note 4·9.5MeCN and 5·16MeCN are isomorphic. Molecules of 5 pack in a square-grid tetragonal array (Fig. S9, ESI†) with large, solvent-filled voids.

#### Use of sodium hydride

##### *p-tert*-Butylcalix[6]arene complex

**Fig. 6:**
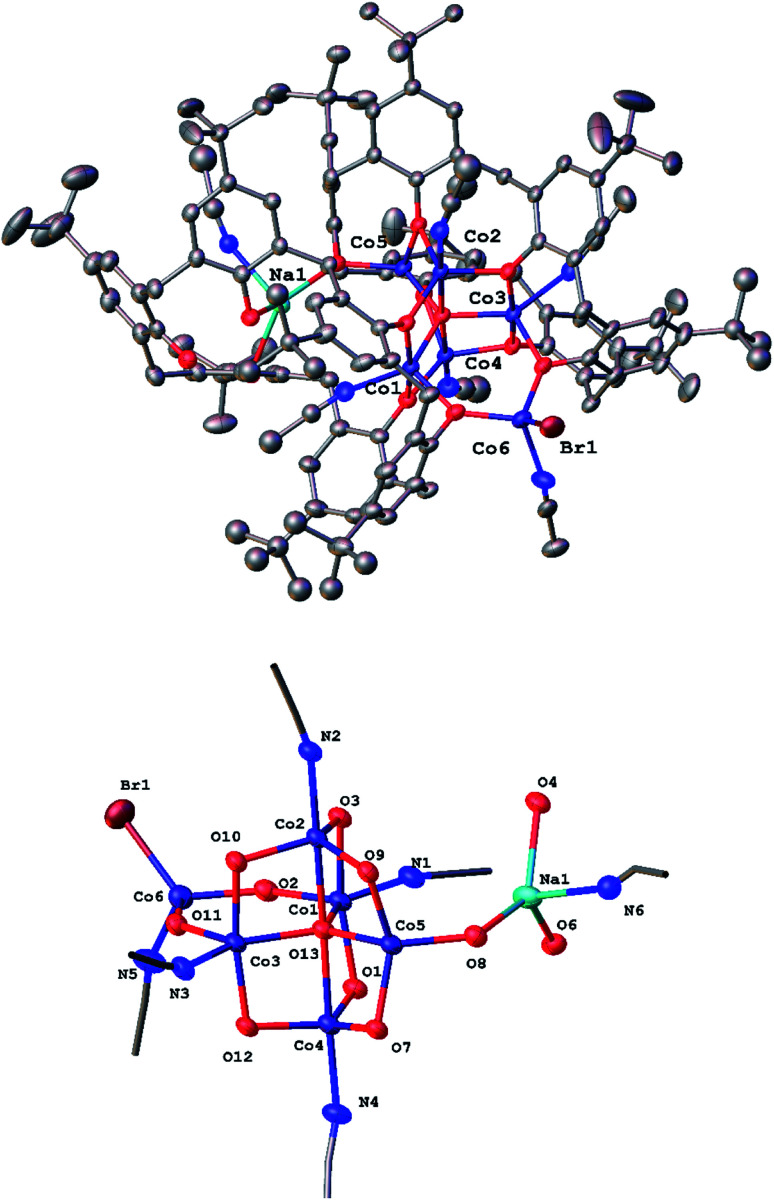
(Top) molecular structure of [Co_6_Na(NCMe)_6_(μ-O)(*p-tert*-butylcalix[6]areneH)_2_Br]·7MeCN (6·7MeCN) with atoms as 50% probability ellipsoids; (bottom) core of 6·7MeCN. For clarity, hydrogen atoms, disorder and acetonitrile molecules are not shown. Selected bond lengths (Å) and angles (°): Co1–O1 2.059(3), Co1–O2 2.021(3), Co1–O3 2.053(3), Co1–O13 1.989(3), Co1–N1 2.069(4), Co2–O3 1.948(3), Co2–O9 1.986(3), Co2–O10 1.956(3), Co2–O13 2.220(2), Co2–N2 2.084(3), Co5–O7 1.952(3), Co5–O8 1.926(3), Co5–O9 1.966(3), Co5–O13 2.016(3), Co6–O2 1.941(3), Co6–O11 1.924(3), Co6–Br1 2.3785(9), Co6–N5 2.066(4); Co1–O1–Co4 98.34(12), Co1–O2–Co6 138.86(15), Co1–O3–Co2 99.06(11), Co3–O13–Co5 122.95(13), Co5–O8–Na1 131.62(14), Co2–O13–Co4 175.30(13).

Given the complexity of the structures of the products obtained from the lithiation reactions, the use of NaH as an entry point was investigated. Various ratios of calix[6 and 8]arenes, NaH and CoBr_2_ were utilized but only on two occasions, one for each calixarene, were crystalline products formed that were suitable for X-ray crystallography. In the case of *p-tert*-butylcalix[6]areneH_6_, addition of six equivalents of NaH followed by the addition of CoBr_2_ (3 equivalents) afforded after work-up (MeCN), blue crystals in *ca*. 70% yield. The single crystal X-ray determination revealed the complex to be [Co_6_Na(NCMe)_6_(μ-O)(*p-tert*-butylcalix[6]areneH)_2_Br]·7MeCN (6·7MeCN). The molecular structure and a view of the core is shown in Fig. 6; selected bond lengths and angles are given in the caption. For an alternative view, see Fig. S10, ESI.†

In 6, 5 cobalt ions surround a central oxide anion in a trigonal bipyramidal arrangement. Each of the equatorial cobalt ions is surrounded by 3 further oxygens from the calixarenes and the effect is to form a central trigonal prismatic core. Attached to this core are 1 further cobalt ion (to which bromide is bound) and a sodium ion. These ions link together the 2 unique calix-6 molecules. MeCN is bound at the metal ions and there is further crystallographically unresolved MeCN modelled using a solvent mask. In terms of charge balance, the 2 Co(ii) centres and sodium cation are off-set by 1 bromide, an oxide, and the 2 mono-protonated calix[6]arene ligands.

##### *p-tert*-Butylcalix[8]arene complex

**Fig. 7:**
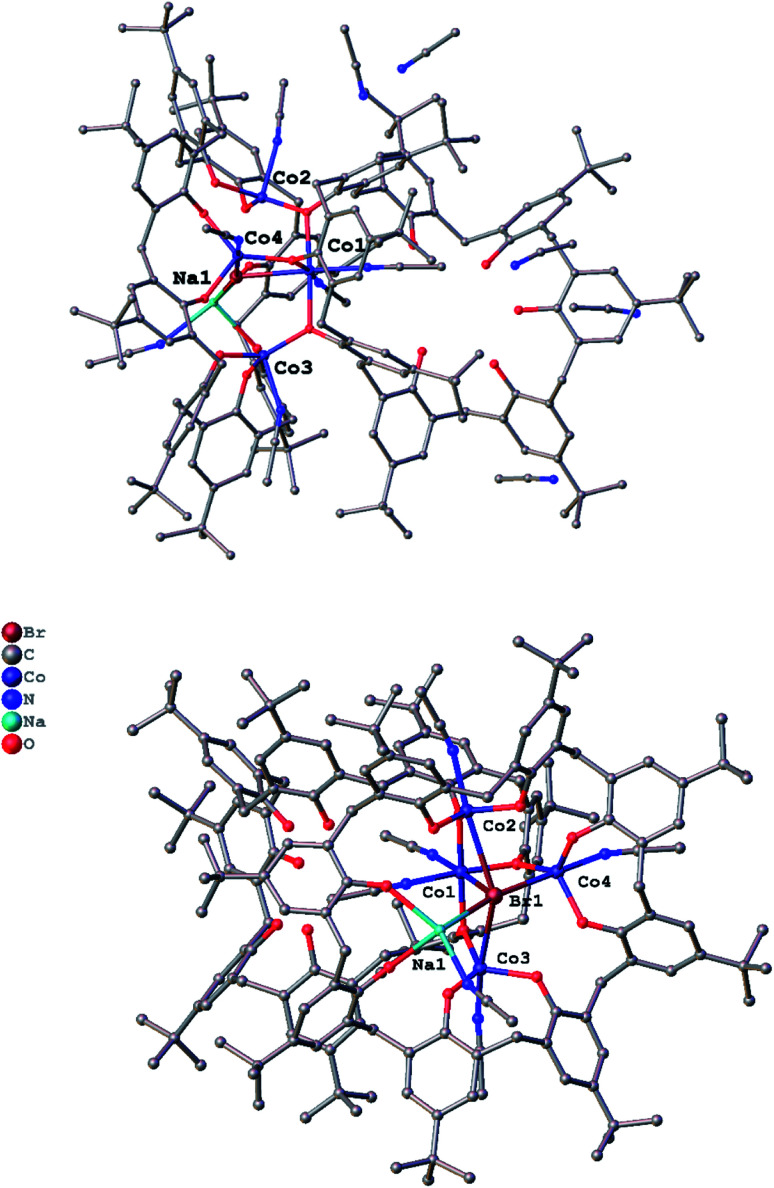
Two views of the molecular structure of [Co_4_Na(NCMe)_4_(μ-O)(*p-tert*-butylcalix[8]arene)(*p-tert*-butylcalix[8]areneH_5_)Br]·13MeCN (7·13MeCN); (bottom) core of 9·6MeCN. For clarity, *t*-butyl groups, hydrogen atoms, disorder and acetonitrile molecules are not shown. For clarity, atoms are drawn as spheres and no hydrogen atoms are illustrated. Selected bond lengths (Å) and angles (°): Co1–O1 2.111(4), Co1–O2 2.023(5), Co1–O3 2.088(4), Co1–N1 2.132(7), Co1–N2 2.070(6), Co1–Br1 2.6425(12), Co2–O1 1.920(4), Co2–O9 1.941(5), Co2–O10 1.974(5), Co2–N3 2.132(7), Co2–Br1 3.0659(13), Co3–O3 1.928(4), Co3–O13 1.987(5), Co3–O14 1.952(5), Co3–N4 2.104(7), Co3–Br1 2.9972(13), Co4–O2 1.944(4), Co4–O11 1.958(4), Co4–O12 1.965(5), Co4–N5 2.099(7), Co4–Br1 2.7491(13); Co1–Br1–Co4 76.88(4), Co3–Br1–Co4 94.13(4), O1–Co1–O3 176.82(17).

For *p-tert*-butylcalix[8]areneH_8_, addition of 8 equivalents of NaH followed by the addition of CoBr_2_ (4 equivalents) afforded, after work-up (MeCN), blue crystals in *ca*. 65% yield. The single crystal X-ray determination revealed the complex to be [Co_4_Na(NCMe)_4_(μ-O)(*p-tert*-butylcalix[8]arene)(*p-tert*-butylcalix[8]areneH_5_)Br]·6MeCN (7·13MeCN). Two views of the molecular structure are shown in Fig. 7, with selected bond lengths and angles given in the caption. For alternative views, see Fig. S11, ESI.†

In 7, there are 4 cobalt centres, 2 calix[8]arene-derived ligands plus a sodium cation and a bromide. One of the macrocycles utilizes all of its oxygens for bonding, whilst the other uses only three (all next to each other). The Co(1) centre is distorted octahedral and possesses the longest Co–O bond lengths [2.023(5) to 2.111(4) Å], whereas the other 3 cobalt centres are trigonal prismatic with shorter Co–O bond lengths [1.920(4) to 1.987(5) Å]. Moreover, we assign oxidations states of +2 to Co(1) and +3 to the other 3 cobalt centres, which in terms of charge accounts for the fully deprotonated calix[8]arene and the other calix[8]arene binding *via* 3 phenolates (Na^+^ and Br^−^ cancel each other out). The 5 non-bonding phenolic protons have been added with a riding model in the structure.

Finally, attempts were also made to access cobalt calix[6 and 8]arenes *via* the use of [Co{N(TMS)_2_}_2_] (TMS = Me_3_Si), however, despite varying the calix : Co ratio, we were unable to obtain products suitable for single crystal X-ray diffraction. We note that the reaction of [Co{N(TMS)_2_}_2_] with *p-tert*-butylcalix[4]areneH_4_ affords [Co_3_(*p-tert*-butylcalix[4]areneOSiMe_3_)_2_THF].^15^

## Electrochemical studies

### Electrochemical analysis in dry electrolyte (0.1 M TEABF_4_ in CH_3_CN) with glassy carbon electrode

**Fig. 8:**
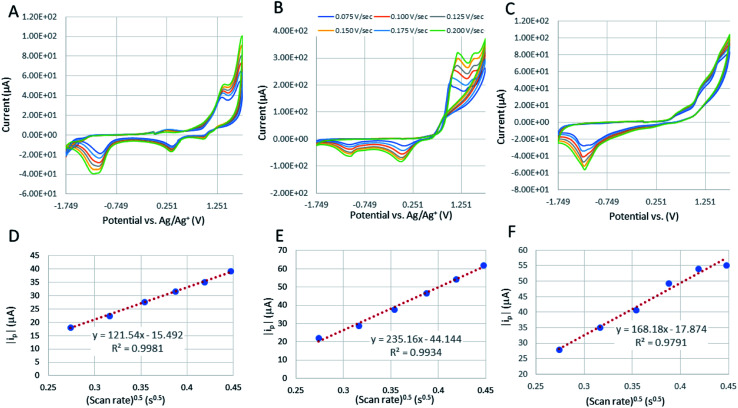
The cyclic voltammograms of a reference HER catalyst (Co(ii)Cl_2_) (A); [Co_6_Na(NCMe)_6_(μ-O)(*p-tert*-butylcalix[6]areneH)_2_Br]·7MeCN (6·7MeCN) (B); and [Co_4_Na(NCMe)_4_(μ-O)(*p-tert*-butylcalix[8]arene)(*p-tert*-butylcalix[8]areneH5)Br]·13MeCN (7·13MeCN) in dry 0.1 M TEABF_4_ (C) in MeCN. CVs recorded at the potential scan rates from 0.05 to 0.225 V s^−1^. The relation between absolute value of cathodic peak current and square root of potential scan rate, *i*_p_ = *f*(√*ν*) is plotted in order to calculate their diffusion coefficient in operating electrolyte for Co(ii)Cl_2_ reference (D) and compounds 6 (E) and 7 (F). Tests are recorded at 1 mM of calixarenes and reference compound.

The cyclic voltammograms of the cobaltocalix[6 and 8]arenes (Fig. 8B and C) reveal several features that we ascribe to metal-based redox events and to the redox activity of the calixarene ligands. The cobaltocalix[6 and 7]arenes screened exhibit similar electrochemical activity with an oxidation of Co(ii) to Co(iii) metal centers that subsequently appear, and the series of anodic peaks starting from 1.1 V, followed by an anodic peak at 1.31 V and corresponding semi-reversible reductions at 1.5 and 0.351 V *vs.* Ag/Ag^+^, respectively. The cathodic peaks at more negative potential represent further reduction of electroactive species present in the calixarene ligand at more negative potential (presumably reaction of quinone to hydroquinone moiety). Notably, with respect to a reference Co(ii)Cl_2_ compound, the Co(ii) transitions observed for cobaltocalixarenes 6 and 7 are very similar. This holds promise that the complexity of a calixarene's structure (*i.e*., bulkiness of ligands) does not influence the charge rate at the metal centers. This is seen as extremely beneficial for achieving an appreciable rate of catalytic activities of the proposed compounds. Although, the redox potential of metal centers can be easily tuned through regulating the electronic structure of ligands, the compounds 6 and 7 demonstrated in Fig. 8B and C (and 1–4) did not experience significant shifts in peak potentials due to the ligand engineering. All oxidations appear quasi-irreversible for cobaltocalix[6 and 8]arenes as well as for the Co(ii)Cl_2_ reference sample, indicating a similar rate of charge exchange, regardless of their chemistries and structure. Notably, 1–4 were excluded from further electrochemical analysis (estimation of a mass transfer and their catalytic activity for electrochemical proton reduction), as they demonstrated electrochemical instability in the operating potential range. This corresponds to the electrochemical activity of phenol moieties of parent calixarenes, resulting in dimerization products or quinone derivatives.^26^ A clear deposit was observed for all compounds 1–4 after prolonged potential scanning. A similar effect was observed for the parent calix[6 and 8]arenes when tested in a similar electrolyte, where the oxidation of phenolic units resulted in the coupling of radical ions with neutral or oxidized molecules of calix[6 and 8]arenes, leading to insoluble oligomeric products that deposit and passivate the electrode.

### Calculation of the diffusion coefficient for [Co_6_Na(NCMe)_6_(μ-O)(*p-tert*-butylcalix[6]areneH)_2_Br]·7MeCN (6·7MeCN) and [Co_4_Na(NCMe)_4_(μ-O)(*p-tert*-butylcalix[8]arene)(*p-tert*-butylcalix[8]areneH_5_)Br]·13MeCN (7·13MeCN)

The correlation of anodic peak current (*i*_p_) as a function of square root of scan rate 
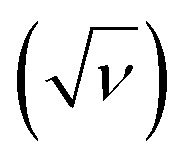
 applied to the Randles–Sevcik (eqn (1)):1
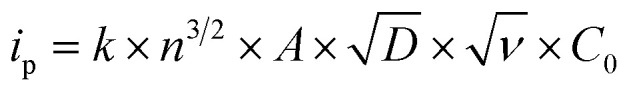
allows the estimation of the diffusion coefficient of the catalyst in the solution (*D* cm^2^ s^−1^), taking into account that *n* is the number of electrons exchanged (*n* = 1 for Co oxidation), *A* is the surface area of the electrode (0.1963 cm^2^), *C* is the molarity of the catalyst solution in mol cm^−3^ (1 mM for all samples), *R* is the gas constant (8.314 J mol^−1^ K^−1^), *T* is the temperature (293.25 K), and *k* is the constant (*k* = 2.69 × 10^5^ C mol^−1^ V^−1/2^). The slope of this linear function is equal to:2
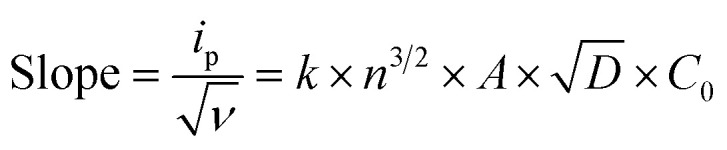


The corresponding slopes of *i*_p_ = *f*(√*ν*) taken from Fig. 8D–F at the oxidation peak of −1.1 V, −1.04 V, and at −1.14 V for Co(ii)Cl_2_, 6, and 7, respectively. The calculated diffusion coefficients (*D*) are 1.13 × 10^−9^, 3.33 × 10^−10^, and 1.01 × 10^−10^ cm s^−1^ for the corresponding calixarenes, as listed previously. The mass transfer coefficients (*D*) are the same order of magnitude for the analyzed calixarenes, which are also comparable with *D* estimated for the Co(ii)Cl_2_ reference, indicating that regardless of the differences in their molecular masses and the complexity of structure, the diffusion rates of active species to the electrode surface are promising and comparable to that of known molecular catalysts tested in similar electrolytes.^27^ The Randles–Sevcik correlations for other calixarene complexes synthesized in this work showed some linearity at lower scan rates (Fig. S12 and S13; Fig. S14† is for 6), however, as they appeared to be unstable under the applied potential, we can only detect a very weak increase in the peak current (*i*_p_) values with increasing scan rate intervals. This is related to the progressing electrode passivation by the decomposition product.

### Catalytic activity for electrochemical proton reduction (hydrogen evolution reaction; HER)

**Fig. 9:**
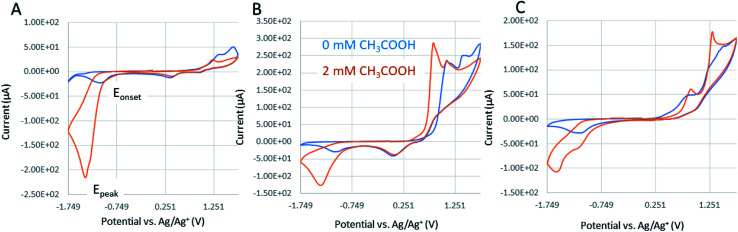
Cyclic voltammograms of 1 mM catalyst in electrolyte for Co(ii)Cl_2_ reference (A); [Co_6_Na(NCMe)_6_(μ-O)(*p-tert*-butylcalix[6]areneH)_2_Br]·7MeCN (6·7MeCN) (B); and [Co_4_Na(NCMe)_4_(μ-O)(*p-tert*-butylcalix[8]arene)(*p-tert*-butylcalix[8]areneH_5_)Br]·13MeCN (7·13MeCN) (C) in dry 0.1 M TEABF_4_ in MeCN at a potential scan rate of 0.1 V s^−1^ recorded in the absence (blue) and presence of the acetic acid substrate (orange; 2 mM equivalent with respect to catalyst concentration).

**Fig. 10:**
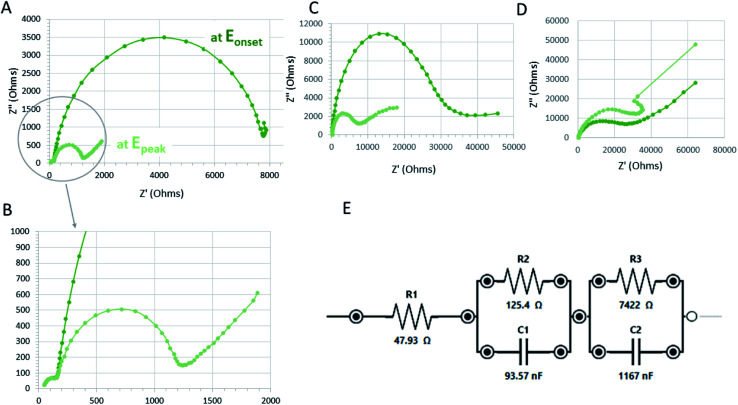
Nyquist plot of 1 mM catalyst in electrolyte for Co(ii)Cl_2_ reference (A) and (B); [Co_6_Na(NCMe)_6_(μ-O)(*p-tert*-butylcalix[6]areneH)_2_Br]·7MeCN (6·7MeCN) (C); [Co_4_Na(NCMe)_4_(μ-O)(*p-tert*-butylcalix[8]arene)(*p-tert*-butylcalix[8]areneH_5_)Br]·13MeCN (7·13MeCN) (D) recorded in the presence of acetic acid substrate (orange; 2 mM equivalent with respect to catalyst concentration). The AC impedance was recorded at constant polarization of *E*_onset_ (dark green) and *E*_peak_ (pale green) as listed in Table 1 for corresponding catalyst. The electrical equivalent circuit used for fitting the impedance is displayed in (E). An example of fitting results to the proposed equivalent circuit with reported error of fitting is included in the ESI (Fig. S16†).

**Fig. 11:**
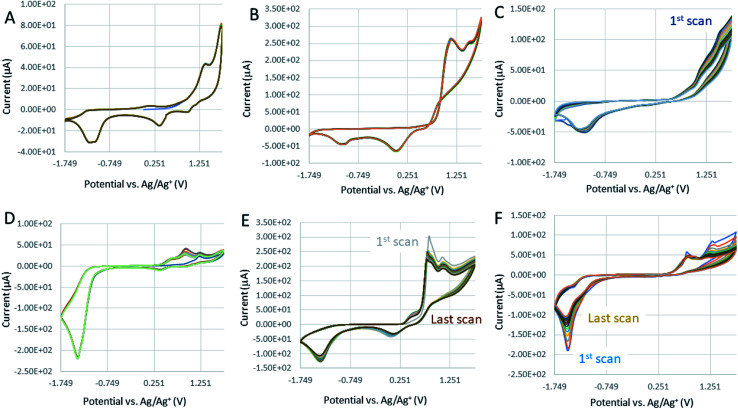
Electrochemical stability tested by cyclic voltammetry (typically 20–50 scans) in the absence of protons (A)–(C) and at 2 mM of acetic acid (D) and (E). Cyclic voltammograms of the reference Co(ii)Cl_2_ (A)–(D); [Co_6_Na(NCMe)_6_(μ-O)(*p-tert*-butylcalix[6]areneH)_2_Br]·7MeCN (6·7MeCN) (B)–(E); and [Co_4_Na(NCMe)_4_(μ-O)(*p-tert*-butylcalix[8]arene)(*p-tert*-butylcalix[8]areneH_5_)Br]·13MeCN (7·13MeCN) (C)–(F) are recorded at 0.15 V s^−1^.

We have attempted to test the calixarene complexes for the electrochemical generation of hydrogen and Fig. 10 demonstrates CV characteristics of the most promising calixarene complexes with respect to Co(ii)Cl_2_ reference catalysts. As the source of the protons, the glacial acetic acid was injected into the electrochemical cell as 0.2 mM aliquots with respect to the molarity of the catalyst (acid concentration was analyzed at 1 mM of catalyst). In order to imply voltametric spectra to predict the kinetics of HER, we have verified the following conditions. Firstly, the catalyst has to demonstrate an unperturbed catalytic response with no-side phenomenon in the presence of the target molecule. This was verified by applying multiple CV scans over the operating potential range in 1 mM catalyst with 2 mM acetic acid and the results are presented in Fig. 11. The results reveal excellent electrochemical stability for the calixarene complexes and the reference material without acid (A)–(C) and acceptable stability in the presence of 2 mM acetic acid ((D)–(F), with slight decrease in signals for compound 7, Fig. 11D). Secondly, we have analyzed whether the electrochemical response for proton reduction is fast as compared with the applied scan rate. This should have given the classical S-shaped CV wave, proving that the proton reduction is independent of the scan rate and the result are shown in Fig. S13† for CVs recorded at 2 mM solution of acetic acid in 1 mM catalyst at scan rates varying from 0.1 to 20 V s^−1^. It is evident that the peak current of proton reduction in the presence of substrate increases with potential scan rate. Hence, the typical foot-to-the-wave analysis of cyclic voltammograms applied to calculate turnover number and frequency (TOF and TON) as well as Tafel-like expression relating TOF to the HER overpotential^28^ of the catalytic process cannot be applied in these studies. As such, we have concluded and demonstrated in Fig. 9 that complexes 6 and 7 (Fig. 9B and C), both demonstrate catalytic activity towards electrochemical proton reduction when compared with Co(ii)Cl_2_ reference (Fig. 9A). This is justified by comparing the magnitude of the current at the potential assigned with the electrochemical proton reduction recorded at the same concentration of acetic acid (2 mM with respect to 1 mM of catalyst). On comparing the *E*_onset_ and *E*_peak_, it can be concluded that the catalytic activity of 6 is very similar to that of Co(ii)Cl_2_, while 7 exhibited a slightly lower peak maximum and current for HER. We have verified that two of the cobalt calixarenes (6 and 7) act as homogenous catalysts for electrochemical proton reduction and report their catalytic overpotential (*η*; mV) with respect to the reference Co(ii)Cl_2_ sample (Fig. 9 and Table 1). Appreciable catalytic rates are achieved at *η* from 14 to 140 mV for the reference catalyst and 6 and 7, and experimental evidence suggests that the catalysis occurs homogeneously. Notably, the onset for the catalytic current is near that reported for proton reduction in similar non-aqueous electrolytes and molecular cobalt electrocatalysts based on trimetallic Co(iii)/Co(ii) cobalt complexes with bridging acyl–alkoxy ligands.^29^

### AC impedance spectroscopy analyzed at constant potentials in the catalytic turnover region

Electrocatalytic proton reduction is further analyzed by impedance spectroscopy where the activity trend is correlated with a charge transfer resistance (*R*_ct_; in this work *R*_ct_ corresponds to resistor *R*_3_ in proposed equivalent circuit; Fig. 10E). By recording the impedance spectrum at onset potential (*E*_onset_) or at the potential of peak maximum these reactions are totally controlled by the kinetics (*i.e.*, the charge transfer). Hence, there is no contribution from diffusion-controlled process as observed in CVs by a scan rate-dependent increase of a peak current for the catalytic proton reduction (Fig. S15†). This diffusion-controlled region can be very well separated in a Nyquist plot as it is represented by a different resistor (Warburg impedance).

Fig. 10A–D represent Nyquist plots recorded at constant potential where the rate of catalytic proton reduction is the highest (within the peak maximum for the proton reduction in CVs, Fig. 9). This is represented by a shape of a depressed semi-circle as shown in Fig. 10. The spectrum is composed of an uncompensated solution resistance (*R*_1_; estimated from the left-side intercept of the first semicircle at the highest frequencies and independent of the applied potential), in series to *R*_1_, there is a resistance of the electrode (*R*_2_, independent of applied potential and estimated from the right-side intercept of the first semicircle), and the most important in this analysis is a charge-transfer resistance (*R*_3_) in parallel connection with C_2_ (a double-layer capacitance at the electrode–electrolyte interface). As observed in Fig. 10C and D (Nyquist plots of compounds 6 and 7), at high-frequencies, *R*_2_ and C_2_ become negligible and its impedance becomes comparable with *R*_1_ (hence the *R*_2_–*C*_1_ element becomes invisible for calixarenes comparing with a separate arc in the spectrum of the reference sample, Fig. 10A and B). In the whole frequency range, the *R*_3_ element (charge transfer resistance) is the only variable affected by the electrode polarization (applied potential *E*_onset_ or *E*_peak_), thus *R*_3_ values serve as the base for making a rational comparison among the studied electrocatalysts on their activity trend. Based on *R*_3_ values (Table 1), we have a rational comparison of a given set catalysts that align well with the trend observed by cyclic voltammetry, from which we can evidence that compound 6 demonstrates proton electrocatalysis similar to that of the Co(ii)Cl_2_ sample (similar peak current magnitude and *R*_3_ resistances) and compound 7 is the weakest and least stable in the presence of acid (order of magnitude higher charge transfer resistance that shows no changes with applied potential as observed in Fig. 10D). The electrochemical degradation of compound 7 in the presence of proton is further confirmed in Fig. 11C.

## Conclusion

In conclusion, reactions between *p-tert*-butylcalix[6 and 8]arenes, lithium reagents (*n*-BuLi or *t*-BuOLi), or the sodium reagent NaH, and CoBr_2_ under different reaction conditions lead to products with complicated molecular structures, which are crystallographically challenging, often with positional disorder. Despite this, the connectivity in the products could sensibly be assigned. A number of these cobaltocalix[6 and 8]arenes have been screened for their electrochemical activity. Results suggest that [Co_6_Na(NCMe)_6_(μ-O)(*p-tert*-butylcalix[6]areneH)_2_Br]·7MeCN (6·7MeCN) is a promising molecular catalyst for electrochemical proton reduction, with a mass transport coefficient, catalytic charge transfer resistance and current magnitude at the catalytic turnover region that are comparable with that of the reference electrocatalyst. With impedance analysis aligned with voltammetry studies, we have attempted to provide insight into the electrochemistry of the cobalt calixarenes. The hope was to encourage other researchers to further explore related systems as electrocatalysts, however the problematic crystallography, typified by the surprising interchangeable positional nature of the cobalt and lithium centres, suggests that such systems are too complex to prove useful in further studies.

## Conflicts of interest

There are no conflicts of interest to declare.

## Supplementary Material

RA-012-D2RA01009G-s001

RA-012-D2RA01009G-s002
