# Supplementary material for: Heterometallic cobalt(ii) calix[6 and 8]arenes: synthesis, structure and electrochemical activity
Source: RSC Adv. 2022 Apr 14;12(19):11672–85. doi: 10.1039/d2ra01009g (PMC9010074; doi:10.1039/d2ra01009g)
Supplement: RA-012-D2RA01009G-s001 [file RA-012-D2RA01009G-s001.pdf]

Electronic Supplementary Material (ESI) for RSC Advances.
This journal is © The Royal Society of Chemistry 2022

Supporting information

Heterometallic cobalt(II) calix[6 and 8]arenes: synthesis, structure and electrochemical activity

Anna Ignaszak,[a*] Nigel Patterson,[a] Connor O'Brien,[a] Allison True,[a] Mark R. J. Elsegood[b] Timothy J. Prior[c] and Carl Redshaw[c*]

[a] *Department of Chemistry, University of New Brunswick, 30 Dineen Drive, Fredericton, NB, E3B 5A3, Canada.*

[b] *Chemistry Department, Loughborough University, Loughborough, Leicestershire, LE11 3TU, UK.*

[c] *Department of Chemistry, University of Hull, Cottingham Road, Hull, HU6 7RX, UK.*

Contents

Figure S1. LCMS of **6**·7MeCN

Figure S2. LCMS of **7**·13MeCN

Figure S3. Alternative view of **1**·15MeCN**.**

Figure S4. Alternative views of **2**·5MeCN·THF**.**

Table S1. Occupancies for Co/Li in **2**·5MeCN·THF.

Figure S5. Alternative view of **3**·28MeCN**.**

Table S2. Occupancies for Co/Li in **3**·28MeCN.

Figure S6. Large channels observed in **3**·28MeCN.

Figure S7. Alternative view of **4**·16MeCN**.**

Table S3. Mixed site occupancies in **4**·16MeCN**.**

Figure S8. Space filling view of **4**·16MeCN.

Figure S9. Alternative views and packing observed in **5**·16.5MeCN

Table S4. Co/Li occupancies in **5**·16.5MeCN.

Figure S10. Alternative view of **6**·7MeCN.

Figure S11. Alternative views of **7**·13MeCN.

Figure S12. Cyclic voltammogram of **1** in dry 0.1 M $TEABF_4$ in MeCN at various potential scan rate.

Figure S13. Cyclic voltammogram of **4** in dry 0.1 M $TEABF_4$ in MeCN at various potential scan rate.

Figure S14. Cyclic voltammogram of **6** in dry 0.1 M $TEABF_4$ in MeCN at various potential scan rate.

Figure S15. The scan rate-dependent evaluation of proton reduction signals at 2 mM CH_3COOH in electrolyte containing 1 mM of the calixarene catalyst.

Figure S16. An example of impedance fitting results (overlay of experimental data with fitting of the proposed equivalent circuit (displayed in Figure 10 E).

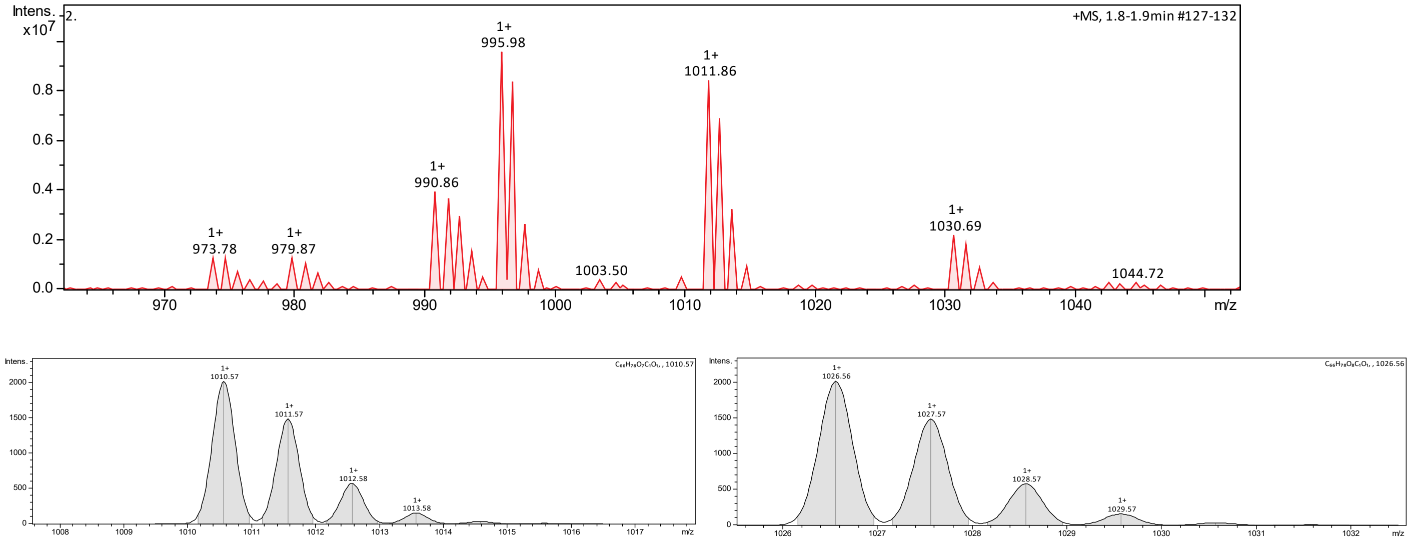

Figure S1. Top: LCMS of **6**·7MeCN (1046 (M – 13MeCN – Br – Na – 5Co – [6]H_2), 1030 (M – 13MeCN – Br – Na – 5Co – O – [6]H_2); bottom: simulated peaks.

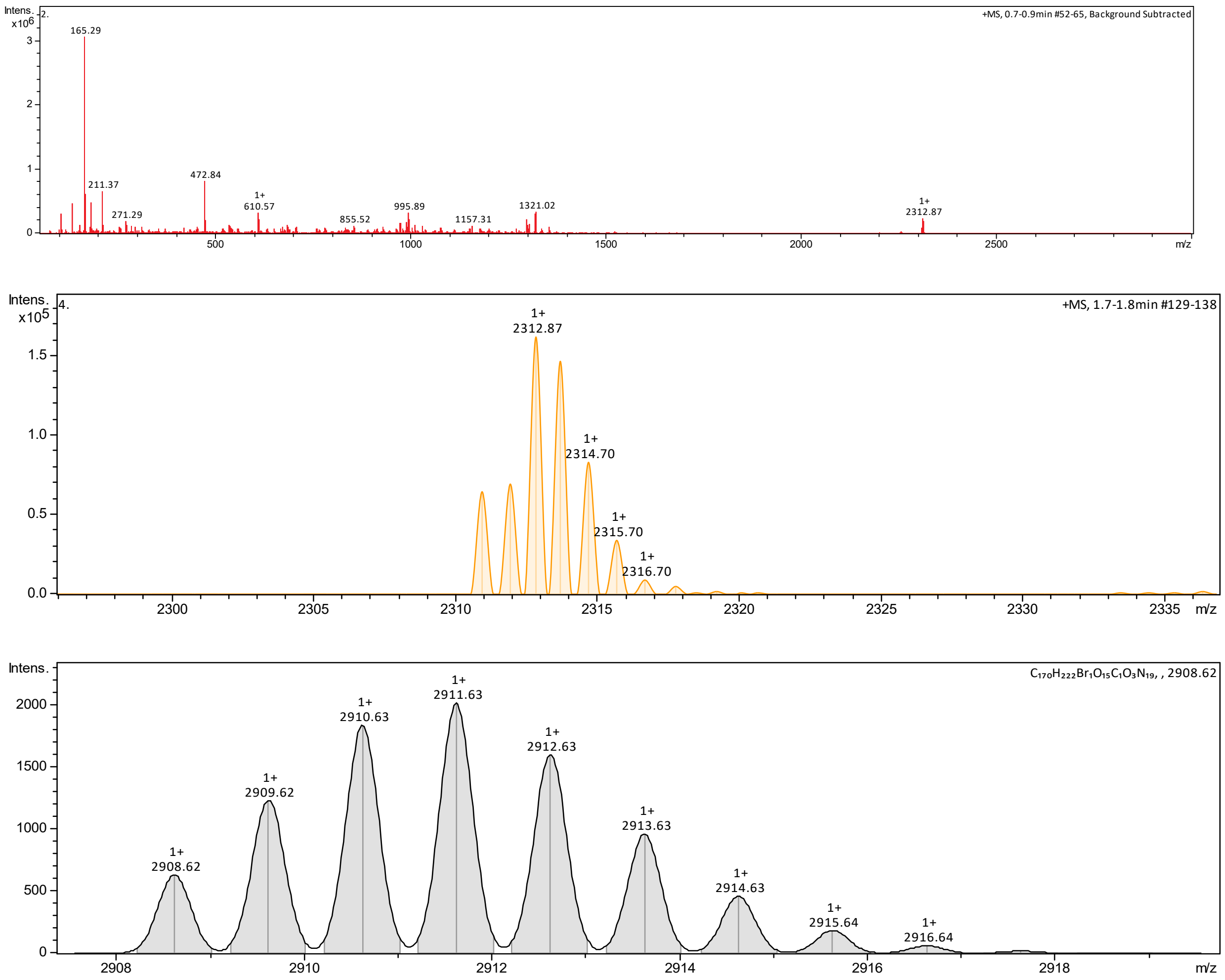

Figure S2. Top/middle: LCMS of **7**·13MeCN (2315 (M – p-*tert*-butylcalix[8]arene – Na – O – Co); bottom: simulated spectrum.

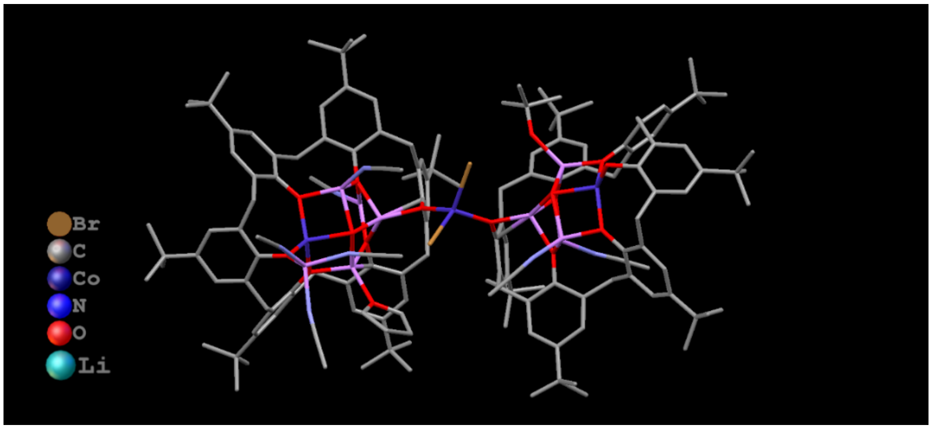

Figure S3. Alternative view of **1**·15MeCN**.**

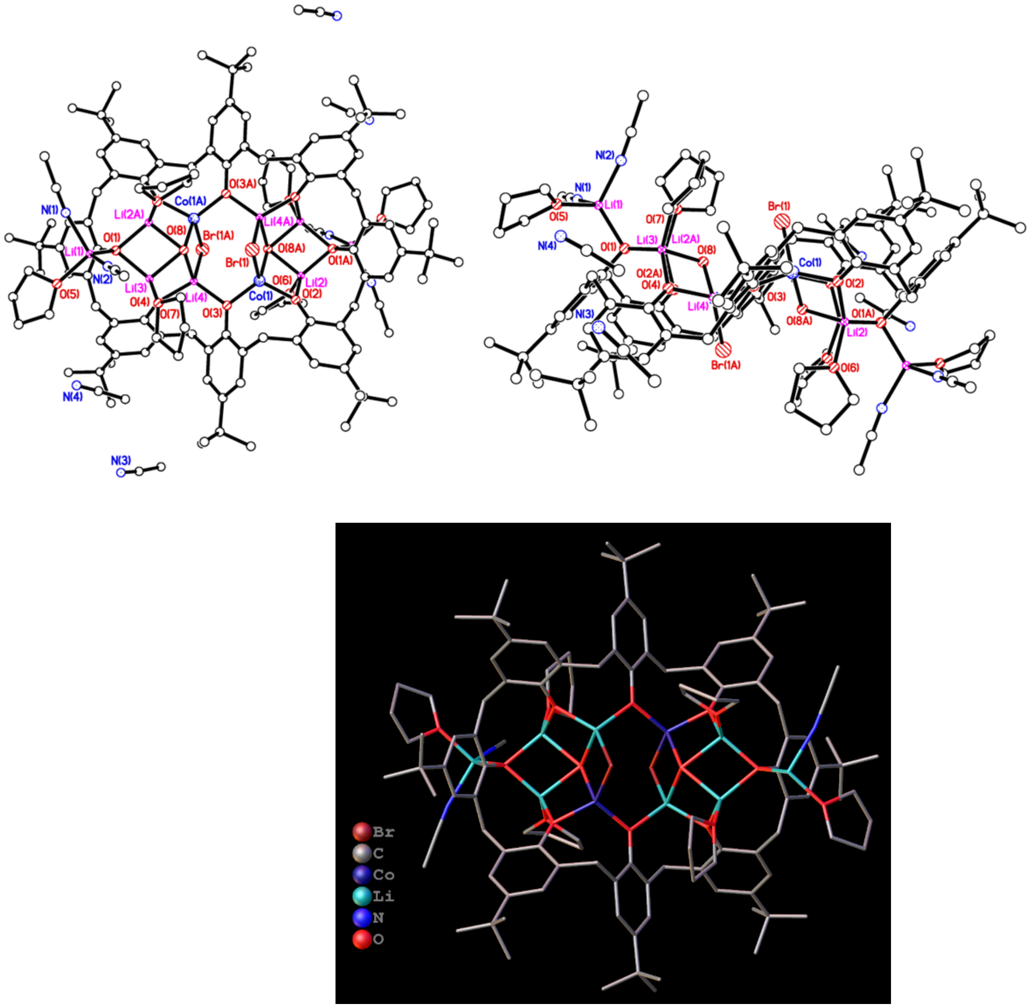

Figure S4. Alternative views of **2**·5MeCN·THF**.**

Table S1. Occupancies for Co/Li in **2**·5MeCN·THF.

	Refined occupancy
Co1<br>Li14X	0.628(3)<br>0.372(3)
Li4<br>Co1X	0.628(3)<br>0.372(3)
Li1	1
Li2	1
Li3	1
Total Co:Li	1: 4

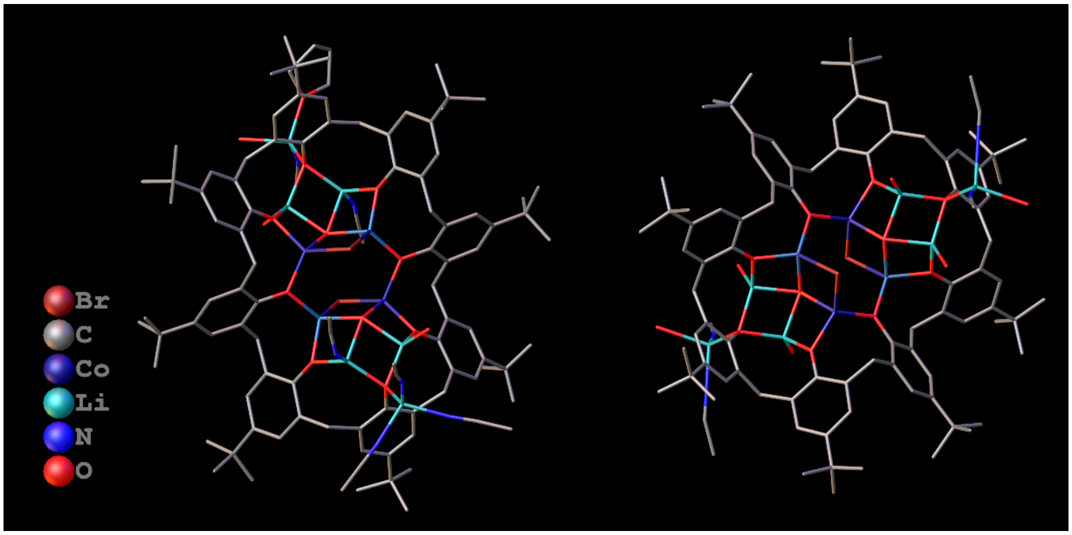

Figure S5. Alternative view of **3**·28MeCN**.**

Metal site occupancies (Co/Li) for **3** were refined freely subject to restraints on the displacement parameters. In this example there was a clear distinction between disordered and not-disordered metal sites. In each case the occupancy of each disordered metal site was refined. Each converged to 0.5 within error. The occupancy of these sites was thus fixed at 0.5 in the final refinement.

Table S2. Occupancies for Co/Li in **3**·28MeCN.

	Occupancy
**Calixarene 1 (10 metal sites) Co_3Li_7**	
Co1	1
Co2<br>Li2A	0.5<br>0.5
Co3	1
Co4<br>Li4A	0.5<br>0.5
Li1	1
Li2	1
Li3	1
Li4	1
Li5	1
Li6	1
**Calixarene 2 (5 unique metal sites, full molecule generated by centre of inversion) $Co_{1.5}Li_{3.5}$; ∴ same as above.**	
Co5<br>Li5A	0.5<br>0.5
Co6	1
Li7	1
Li8	1
Li9	1

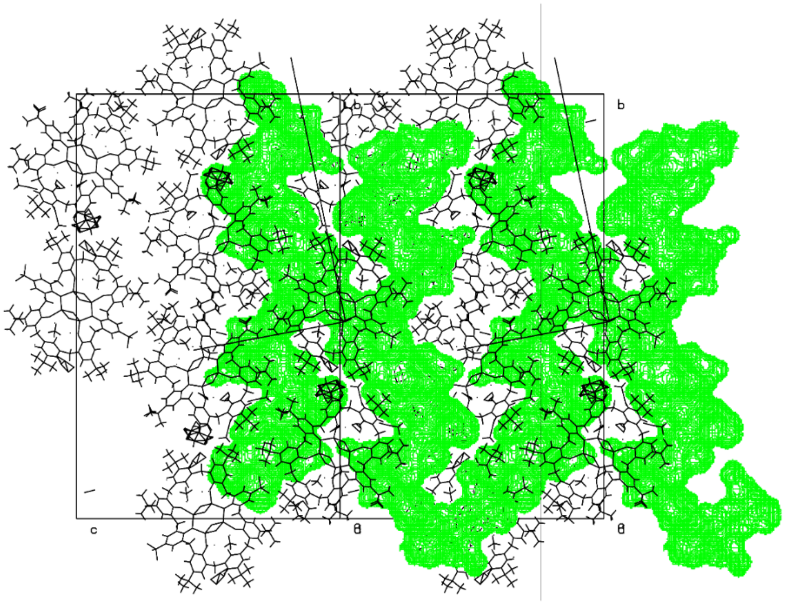

Figure S6. Large channels observed in **3**·28MeCN.

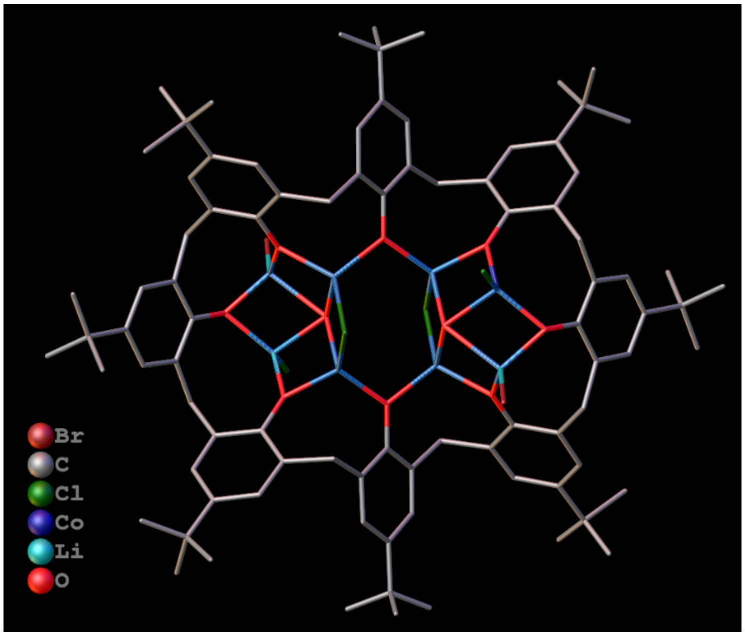

Figure S7. Alternative view of **4**·16MeCN**.**

Table S3. Mixed site occupancies in **4**·16MeCN**.**

	Refined occupancy
Co1<br>Li1	0.596(4)<br>0.404(4)
Co2<br>Li2	0.392(4)<br>0.608(4)
Co3<br>Li3	0.311(4)<br>0.689(4)
Co4<br>Li4	0.104(2)<br>0.896(2)
Total Co:Li	1.403: 2.597
Br1<br>Cl1	0.630(6)<br>0.370(6)
Br2<br>Cl2	0.058(10)<br>0.942(10)
Total Br:Cl	0.688: 1.312

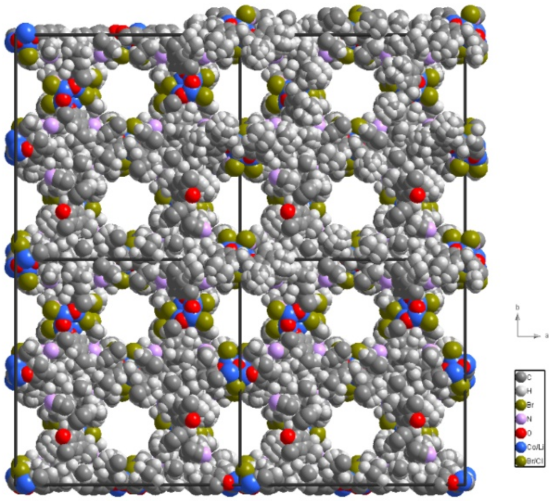

Figure S8. Space filling view of **4**·16MeCN.

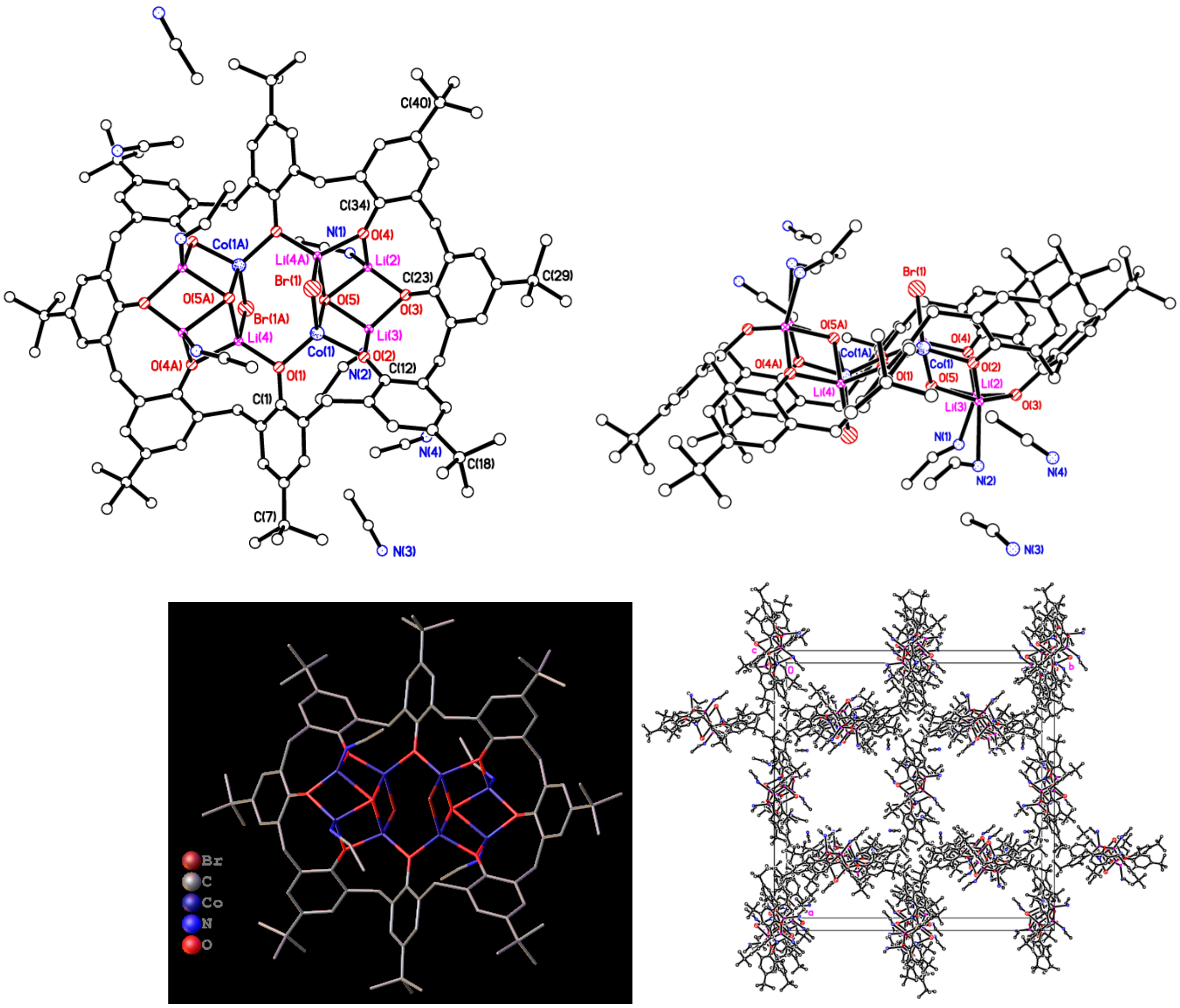

Figure S9. Alternative views and packing observed in **5**·16.5MeCN

Table S4. Co/Li occupancies in **5**·16.5MeCN.

	Refined ratio
Co1/Li1	0.500 : 0.500(3)
Co2/Li2	0.072 : 0.928(4)
Co3/Li3	0.150 : 0.850(3)
Co4/Li4	0.476 : 0.524(3)
Total Co:Li	1.198: 2.802

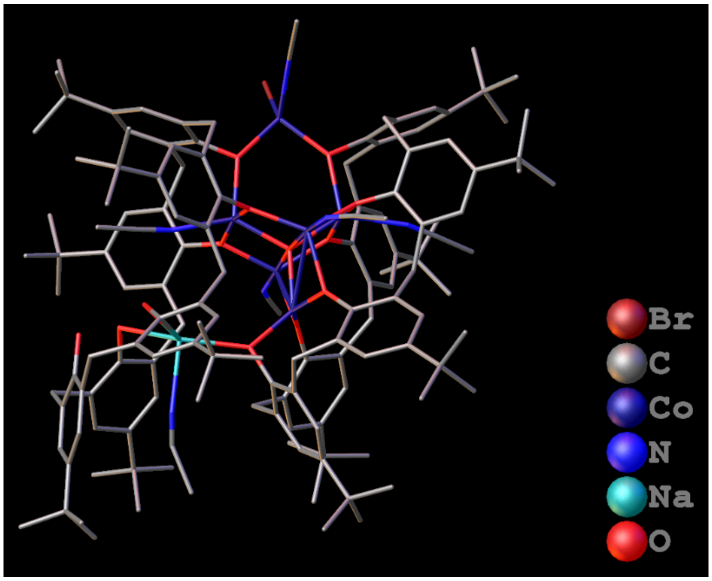

Figure S10. Alternative view of **6**·7MeCN.

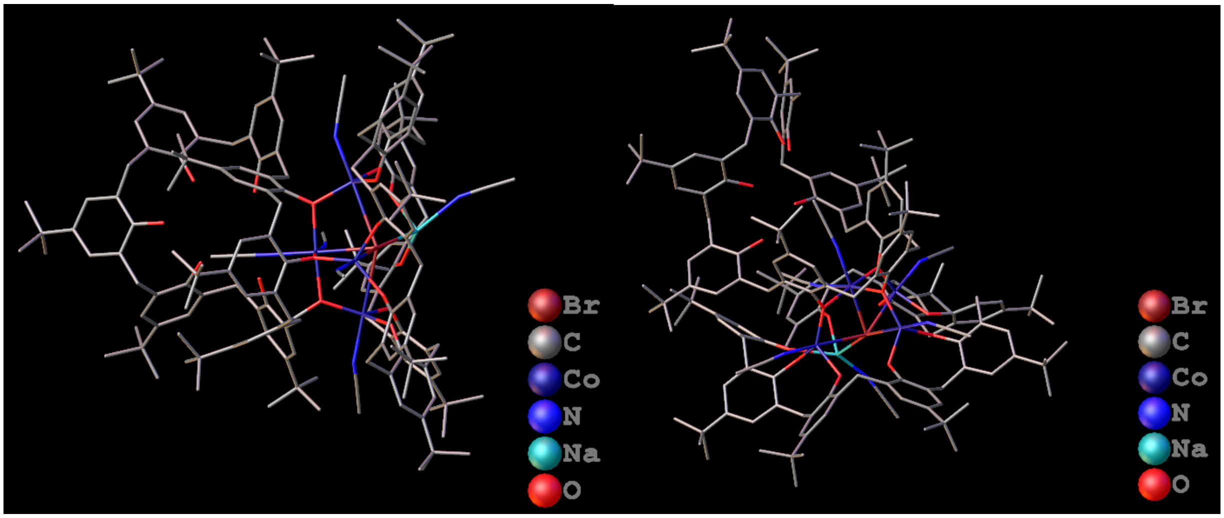

Figure S11. Alternative views of **7**·13MeCN.

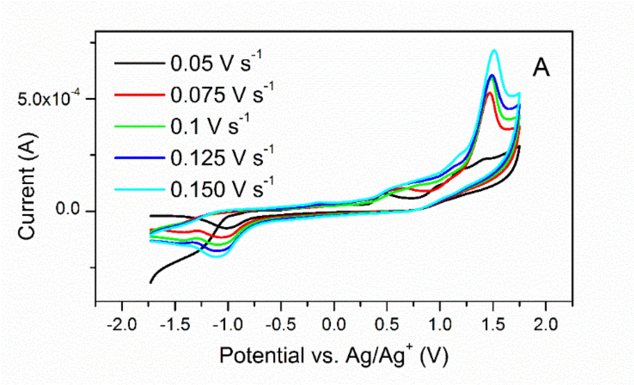

Figure S12. Cyclic voltammogram of **1** in dry 0.1 M $TEABF_4$ in MeCN at various potential scan rate.

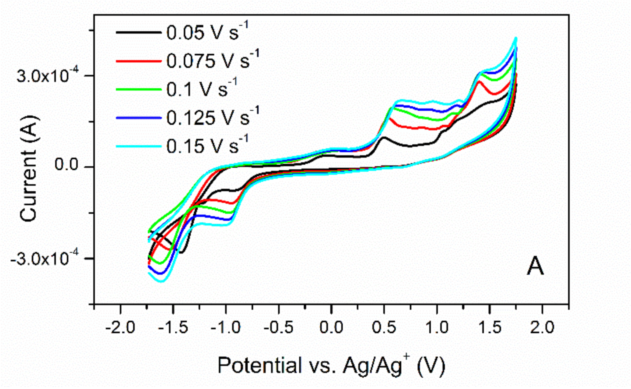

Figure S13. Cyclic voltammogram of **6** in dry 0.1 M $TEABF_4$ in MeCN at various potential scan rate.

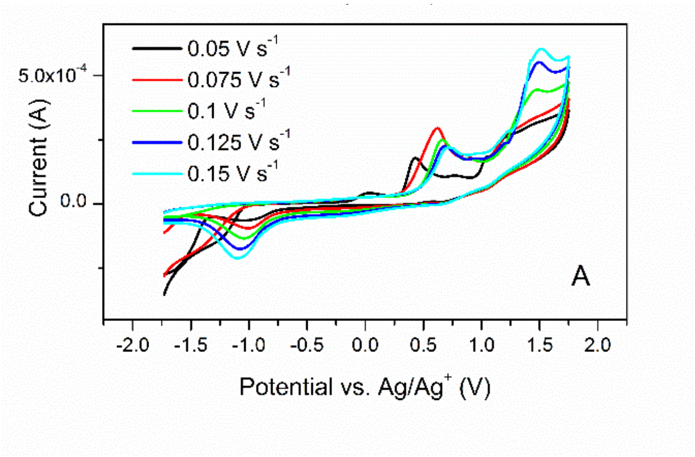

Figure S14. Cyclic voltammogram of **6** in dry 0.1 M $TEABF_4$ in MeCN at various potential scan rate.

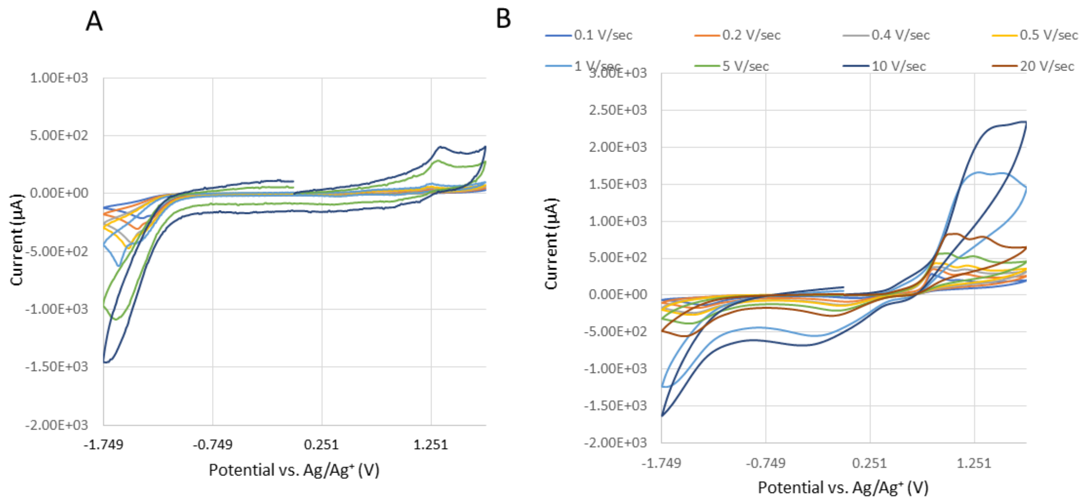

Figure S15. The scan rate-dependent evaluation of proton reduction signals at 2 mM CH_3COOH in electrolyte containing 1 mM of the calixarene catalyst.

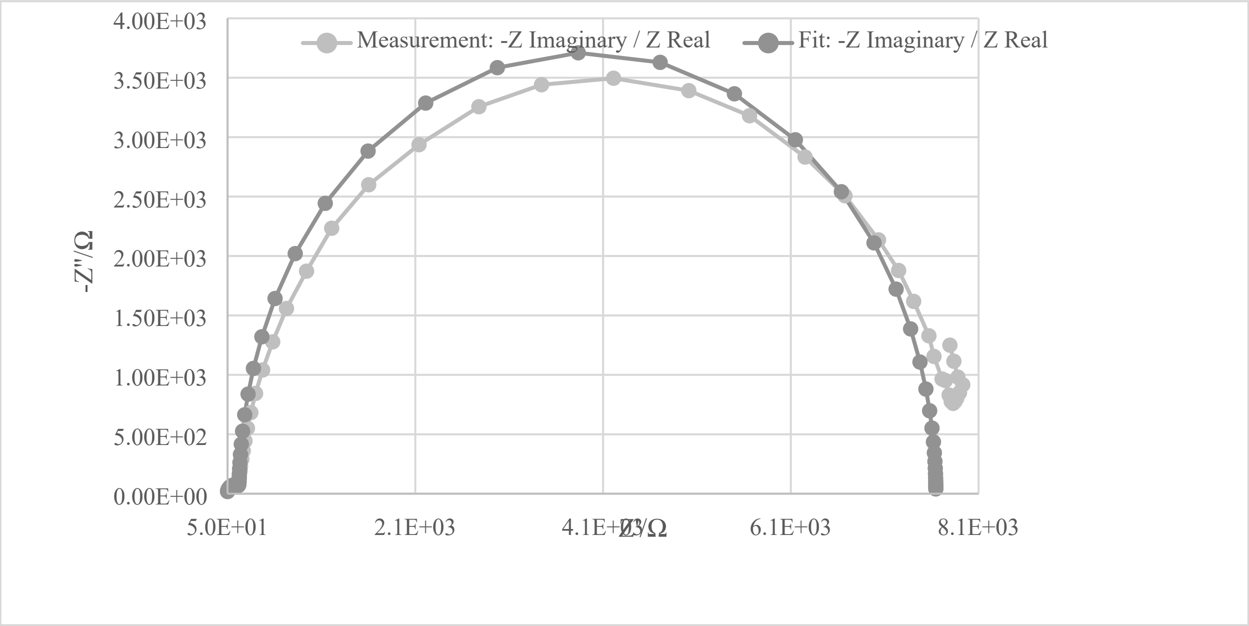

Circuit:	R(RC)(RC)	Title:	PGScan at 59 freqs - E=- 1.000 V	Measurement :	Impedance Spectroscopy	
Fixed	Element	Fitted Value	Min Value	Max Value	Unit	Error%
False	R 1	47.93	1.00E-6	1.00E+12	Ω	5.171
False	R 2	125.4	1.00E-6	1.00E+12	Ω	3.879
False	C 1	9.357E-8	1.00E-12	1.00E-3	F	6.645
False	R 3	7422	1.00E-6	1.00E+12	Ω	1.900
False	C 2	1.167E-6	1.00E-12	1.00E-3	F	2.294
	Chi-Squared:	0.0077	Iterations:	1		

Figure S16. An example of impedance fitting results (overlay of experimental data with fitting of the proposed equivalent circuit (displayed in Figure 10 E).
